# SynthEHR-eviction: enhancing eviction SDoH detection with LLM-augmented synthetic EHR data

**DOI:** 10.1038/s41746-026-02473-0

**Published:** 2026-02-27

**Authors:** Zonghai Yao, Youxia Zhao, Avijit Mitra, David A. Levy, Emily Druhl, Jack Tsai, Hong Yu

**Affiliations:** 1 Center for Healthcare Organization and Implementation Research, VA Bedford Health Care, Bedford, MA USA; 2 Manning College of Information and Computer Sciences, Umass Amherst, Amherst, MA USA; 3 Department of Medicine, University of Massachusetts Medical School, Worcester, MA USA; 4 National Center on Homelessness among Veterans, VA Homeless Programs Office, Washington, DC USA; 5 School of Public Health, University of Texas Health Science Center at Houston, Houston, TX USA; 6 Department of Psychiatry, Yale University School of Medicine, New Haven, CT USA; 7 Miner School of Computer and Information Sciences, UMass Lowell, Lowell, MA USA

**Keywords:** Computational models, Data publication and archiving, Computer science

## Abstract

Eviction is a significant yet understudied social determinants of health (SDoH), linked to housing instability, unemployment, and mental health. While eviction appears in unstructured electronic health records (EHRs), it is rarely coded in structured fields, limiting downstream applications. We introduce SynthEHR-Eviction, a scalable pipeline that adapts and integrates human-in-the-loop annotation, automated prompt optimization (APO), and reasoning-augmented fine-tuning for low-resource eviction-related SDoH extraction from clinical notes. Using this pipeline, we created a large public eviction-related SDoH dataset to date, comprising 14 fine-grained categories. Fine-tuned LLMs (e.g., Qwen2.5, LLaMA3) trained on SynthEHR-Eviction achieved Macro-F1 scores of 88.8% (eviction) and 90.3% (other SDoH) on human validated data, outperforming GPT-4o-APO (87.8%, 87.3%), GPT-4o-mini-APO (69.1%, 78.1%), and BioBERT (60.7%, 68.3%), while enabling cost-effective deployment across various model sizes. The pipeline reduces annotation effort by over 80%, accelerates dataset creation, enables scalable eviction detection, and generalizes to other information extraction tasks.

## Introduction

In modern healthcare systems, advancing individualized, whole-person care and promoting access to comprehensive healthcare and social services have become key goals of global healthcare movements^1,2^. Beyond clinical indicators, environmental and social actors can also greatly influence health outcomes. The World Health Organization (WHO) has recognized the wide-ranging influence of social determinants of health (SDoH) as “the conditions in which people are born, grow, live, work, and age"^3^ and notes that they contribute to as much as 80–90% of health outcomes^4^. Accurately and systematically integrating SDoH into clinical decision-making is a critical step toward achieving personalized care, promoting health and well-being, and enhancing the effectiveness of public health interventions^5,6^. For example, individuals who are unstably housed may live in precarious, unsafe conditions that can affect their physical and mental health^7,8^. Lack of transportation can affect access to healthcare and daily necessities, such as healthy foods^9,10^.

Yet, in current electronic health records (EHRs), SDoH information is often embedded within unstructured free-text clinical notes, lacking systematic, structured documentation^11^. This results in “information silos", where crucial social risk factors are underutilized in real-world healthcare delivery, limiting clinicians’ ability to assess patients’ broader health risks and restricting healthcare institutions and policymakers from designing proactive, targeted intervention strategies^12^.

Among various categories of SDoH, eviction is a highly impactful but long-overlooked factor affecting patient health. Eviction can trigger a chain reaction of adverse outcomes, including housing instability, unemployment, homelessness, mental health issues, and long-term poverty^13,14^. Its impact extends beyond individuals, affecting entire communities and exacerbating social inequality^15^. Studies have shown that marginalized populations, such as racial minorities, women, and low-income renters, face a significantly higher risk of eviction^16^. Particularly during the Coronavirus Disease-2019 (COVID-19) pandemic, eviction was directly associated with over 430,000 additional cases and more than 10,000 deaths in the United States, prompting the CDC to enact a federal eviction moratorium between 2020 and 2021^17^. Despite its profound public health implications, the current healthcare system’s ability to identify and address eviction-related risks remained severely limited. Mainstream public EHR datasets, such as MIMIC-III^18^ and MIMIC-IV^19^, contain extremely sparse documentation of eviction-related information, with relevant terms appearing in fewer than 1 in 1000 records. Furthermore, standardized coding systems, such as relying on the International Classification of Diseases, Version 10 (ICD-10), lack specific codes for eviction, contributing to fragmented and underutilized eviction data in clinical practice^20^. Unlocking and systematically incorporating eviction-related SDoH data can transform healthcare delivery by enabling comprehensive health risk assessments, integrating social work, housing support, and psychological interventions into patient care workflows, and providing real-time, high-quality data to support targeted public health interventions, particularly benefiting marginalized populations and advancing accessible healthcare and social service systems.

In response to these challenges and transformative demands, we develop a novel, scalable information extraction (IE) pipeline tailored for eviction-related SDoH annotation. Unless otherwise specified, throughout this paper, “SDoH classes” primarily refers to eviction-related SDoH classes, supplemented by Tier 1 and Tier 2 ICD-10 Z-codes, rather than a comprehensive SDoH representation. IE has long been a central goal in clinical NLP^21^, with traditional approaches relying on rule-based extraction^22^ or small model fine-tuning methods^23^ that are time-consuming, difficult to scale, and often institution-specific. Recent advances in large language models (LLMs) have shown promise in scaling IE development through synthetic data generation and prompt-based learning^24^. These techniques can significantly reduce annotation costs and improve generalization, yet their use in complex, underrepresented SDoH domains remains limited^11,25,26^. Eviction, as a high-impact but rarely modeled SDoH^27^, exemplifies the need for clinically grounded, low-cost annotation solutions. Specifically, in many SDoH and clinical annotation settings, labeled data are severely scarce, making it difficult to directly train reliable annotation models. We therefore design our pipeline as a unified, two-stage end-to-end solution that integrates clinically grounded synthetic data generation with a reusable multi-class annotation framework: the former addresses data scarcity by providing controlled training examples, while the latter operationalizes these data for downstream labeling and dataset construction.

We introduce *SynthEHR-Eviction*, a scalable and modular pipeline that integrates LLM-based data augmentation, automated prompt optimization (APO)^28^, and human-in-the-loop (HITL) validation to support large-scale, consistent annotation. During the augmentation phase, expert feedback is incorporated to refine prompts and balance label distributions. While APO and reasoning supervision have been studied previously, our contribution is to operationalize and adapt them into a unified, clinically grounded workflow for eviction-related SDoH, enabling reliable multi-class annotation and model training under severe label scarcity. For annotation, we adopt the DSPy framework^29^, which enables chain-of-thought (CoT) annotation, automated reasoning trace generation, and fine-grained label refinement. The pipeline involves two iterative stages: First, the augmenter generates new examples for underrepresented categories (see Supplementary Table S6 and Supplementary Table S7 for class definitions); Second, the annotator verifies and labels the generated notes while producing structured reasoning. Compared to full manual rewriting and annotation, this workflow reduces human labor by over 80% while maintaining high data quality and interpretability. Moreover, its modular structure generalizes to other SDoH domains such as food insecurity, utility shutoff, or intimate partner violence^30^. A concrete illustration of how the pipeline can be extended to other SDoH domains is provided in Supplementary Information (Illustrative Extension to Other SDoH Domains).

Using this pipeline, we constructed the SynthEHR-Eviction dataset, the largest publicly available dataset of SDoH related to evictions to date. It covered 14 carefully defined SDoH categories, including fine-grained eviction statuses such as “Eviction Absent," “Eviction Pending," and “Mutual Rescission History" (see Supplementary Table S6) as well as related ICD-10 Z.59 categories like housing instability and homelessness (see Supplementary Table S7). In total, the dataset comprises 8000 synthetic training and development instances, alongside 616 expert-annotated test instances that encompass both synthetic and real-world clinical notes, drawn from sources such as MIMIC-IV^19^ and PMC-Patients^31^. This publicly available dataset not only fills a critical gap in eviction-related benchmarks but also demonstrates strong adaptability by enabling the fine-tuning of open-source LLMs for downstream use. It offers a practical and efficient foundation for building SDoH recognition systems and integrating social care into routine clinical workflows.

## Results

Our experimental results demonstrate several key findings across different classification tasks.

### Step 1: main results for eviction mention detection via binary classification

As shown in Table 1, the binary classification task of distinguishing between eviction and non-eviction instances was relatively straightforward across all model architectures. GPT-4o, when equipped with our APO pipeline, consistently achieved the highest F1 scores across all datasets: 0.939 on Synth, 0.938 on MIMIC, and 0.947 on PMC, with an overall average of 0.941. This clearly outperformed the vanilla GPT-4o without APO (average: 0.859), highlighting the benefit of its self-prompt optimization. In contrast, GPT-4o-mini showed no clear gain from APO, suggesting that this smaller LLM may be less capable of benefiting from its self-reflective prompt adjustments for this task.

**Table 1:** Performance comparison of various models across all steps and datasets

Performance results of various models for step 1: binary classification (this is a balanced dataset, so we only report Micro-F1 here)				
**Models**	**Synth**	**Mimic**	**PMC**	**Avg**
GPT-4o-mini	0.761 (0.757-0.764) p < 0.05	0.735 (0.732-0.739) p < 0.05	0.889 (0.879-0.900) p < 0.05	0.795 (0.790-0.801) p < 0.05
GPT-4o-mini-APO	0.796 (0.781-0.811) p < 0.05	0.706 (0.653-0.759) p < 0.05	0.876 (0.860-0.892) p < 0.05	0.793 (0.770-0.816) p < 0.05
GPT-4o	0.880 (0.867-0.894) p < 0.05	0.860 (0.847-0.872) p < 0.05	0.839 (0.826-0.851) p < 0.05	0.859 (0.850-0.869) p < 0.05
SynthEHR-Eviction (bert_base_cased)	0.976 (0.961-0.991) p < 0.01	0.821 (0.780-0.861) p < 0.05	0.857 (0.836-0.878) p < 0.05	0.885 (0.870-0.899) p < 0.05
SynthEHR-Eviction (biobert-v1.1)	**0.987(0.979−0.995)_p<0.01**	0.916 (0.890-0.942)	0.892 (0.854-0.930) p < 0.05	0.931 (0.916-0.947)
SynthEHR-Eviction (Bio_ClinicalBERT)	0.978 (0.960-0.995) p < 0.01	0.756 (0.706-0.806) p < 0.05	0.805 (0.718-0.893) p < 0.05	0.846 (0.813-0.880) p < 0.05
SynthEHR-Eviction (LLama-3.1-8B-FT)	0.966 (0.956-0.976) p < 0.01	0.866 (0.850-0.882) p < 0.05	0.891 (0.875-0.907) p < 0.05	0.908 (0.897-0.919) p < 0.05
SynthEHR-Eviction (LLama-3.2-3B-FT)	0.934 (0.929-0.938)	0.868 (0.858-0.877) p < 0.05	0.824 (0.813-0.834) p < 0.05	0.875 (0.871-0.879) p < 0.05
SynthEHR-Eviction (Qwen2.5-7B-FT)	0.963 (0.955-0.981) p < 0.01	0.912 (0.904-0.920) p < 0.05	0.891 (0.882-0.900) p < 0.05	0.922 (0.918-0.926)
SynthEHR-Eviction (Qwen2.5-3B-FT)	**0.987(0.976−0.997)_ p<0.001**	0.850 (0.840-0.859) p < 0.05	0.834 (0.820-0.848) p < 0.05	0.890 (0.884-0.896) p < 0.05
GPT-4o-APO (baseline)	0.939 (0.912-0.965)	**0.938(0.926−0.949)**	**0.947(0.936−0.958)_**	**0.941(0.931−0.951)_**

Performance results of various models for step 2: eviction multi-class (Macro-F1 shown above, Micro-F1 shown below)				
**Models**	**Synth**	**Mimic**	**PMC**	**Avg**
GPT-4o-mini	0.337 (0.331-0.343) p < 0.05	0.238 (0.217-0.260) p < 0.05	0.243 (0.217-0.269) p < 0.05	0.273 (0.263-0.283) p < 0.05
GPT-4o-mini-APO	0.760 (0.696-0.823) p < 0.05	0.672 (0.652-0.692) p < 0.05	0.641 (0.547-0.735) p < 0.05	0.691 (0.640-0.742) p < 0.05
GPT-4o	0.670 (0.629-0.711) p < 0.05	0.521 (0.502-0.539) p < 0.05	0.352 (0.302-0.403) p < 0.05	0.515 (0.489-0.540) p < 0.05
SynthEHR-Eviction (bert_base_cased)	0.806 (0.790-0.822) p < 0.05	0.784 (0.734-0.834) p < 0.05	0.384 (0.248-0.519) p < 0.05	0.658 (0.604-0.712) p < 0.05
SynthEHR-Eviction (biobert-v1.1)	0.811 (0.792-0.831) p < 0.05	0.668 (0.585-0.752) p < 0.05	0.342 (0.300-0.384) p < 0.05	0.607 (0.568-0.646) p < 0.05
SynthEHR-Eviction (Bio_ClinicalBERT)	0.790 (0.762-0.818) p < 0.05	0.658 (0.630-0.686) p < 0.05	0.346 (0.321-0.371) p < 0.05	0.598 (0.584-0.611) p < 0.05
SynthEHR-Eviction (LLama-3.1-8B-FT)	0.881 (0.820-0.942)	0.935 (0.907-0.962) p < 0.01	**0.848(0.821−0.876)_**	**0.888(0.872−0.904)_**
SynthEHR-Eviction (LLama-3.2-3B-FT)	0.909 (0.895-0.922)	0.812 (0.665-0.958)	0.801 (0.774-0.828)	0.840 (0.784-0.897)
SynthEHR-Eviction (Qwen2.5-7B-FT)	**0.920(0.903−0.938)_**	**0.939(0.933−0.944)_p<0.001**	0.804 (0.767-0.842)	**0.888(0.869−0.907)_**
SynthEHR-Eviction (Qwen2.5-3B-FT)	0.889 (0.824-0.954)	0.850 (0.827-0.873)	0.825 (0.745-0.905)	0.855 (0.824-0.886)
GPT-4o-APO (baseline)	0.918 (0.912-0.925)	0.878 (0.846-0.911)	0.836 (0.750-0.923)	0.878 (0.842-0.914)
GPT-4o-mini	0.504 (0.496-0.512) p < 0.05	0.357 (0.331-0.383) p < 0.05	0.438 (0.410-0.465) p < 0.05	0.433 (0.421-0.444) p < 0.05
GPT-4o-mini-APO	0.840 (0.791-0.889) p < 0.05	0.772 (0.752-0.792) p < 0.05	0.681 (0.609-0.754) p < 0.05	0.764 (0.722-0.807) p < 0.05
GPT-4o	0.754 (0.741-0.767) p < 0.05	0.583 (0.567-0.600) p < 0.05	0.502 (0.459-0.545) p < 0.05	0.613 (0.595-0.631) p < 0.05
SynthEHR-Eviction (bert_base_cased)	0.810 (0.795-0.825) p < 0.05	0.880 (0.842-0.918)	0.444 (0.285-0.603) p < 0.05	0.711 (0.647-0.775) p < 0.05
SynthEHR-Eviction (biobert-v1.1)	0.829 (0.790-0.868) p < 0.05	0.745 (0.700-0.790) p < 0.05	0.434 (0.289-0.579) p < 0.05	0.669 (0.603-0.736) p < 0.05
SynthEHR-Eviction (Bio_ClinicalBERT)	0.791 (0.765-0.818) p < 0.05	0.775 (0.730-0.820) p < 0.05	0.390 (0.343-0.437) p < 0.05	0.652 (0.623-0.682) p < 0.05
SynthEHR-Eviction (LLama-3.1-8B-FT)	0.906 (0.888-0.923)	0.925 (0.909-0.941) p < 0.01	**0.861(0.834−0.888)_**	**0.897(0.882−0.913)_**
SynthEHR-Eviction (LLama-3.2-3B-FT)	0.908 (0.896-0.921)	0.882 (0.848-0.916)	0.824 (0.796-0.853)	0.871 (0.849-0.893)
SynthEHR-Eviction (Qwen2.5-7B-FT)	**0.921(0.905−0.938)_**	**0.938(0.932−0.945)_**p<0.001	0.824 (0.789-0.860)	0.895 (0.877-0.912)
SynthEHR-Eviction (Qwen2.5-3B-FT)	0.911 (0.901-0.921)	0.853 (0.832-0.875) p < 0.05	0.857 (0.801-0.914)	0.874 (0.856-0.892)
GPT-4o-APO (baseline)	0.909(0.886-0.931)	0.883(0.856-0.911)	0.868(0.806-0.929)	0.886(0.858-0.915)

Performance results of various models for step 3: non-eviction multi-class (Macro-F1 shown above, Micro-F1 shown below)				
**Models**	**Synth**	**Mimic**	**PMC**	**Avg**
GPT-4o-mini	0.830 (0.823-0.837) p < 0.05	0.672 (0.666-0.678) p < 0.05	0.645 (0.586-0.704) p < 0.05	0.716 (0.696-0.735) p < 0.05
GPT-4o-mini-APO	0.881 (0.875-0.887)	0.737 (0.716-0.757) p < 0.05	0.724 (0.695-0.754) p < 0.05	0.781 (0.771-0.791) p < 0.05
GPT-4o	0.850 (0.838-0.862) p < 0.05	0.795 (0.769-0.820) p < 0.05	0.746 (0.713-0.779) p < 0.05	0.797 (0.782-0.812) p < 0.05
SynthEHR-Eviction (bert_base_cased)	0.865 (0.858-0.873) p < 0.05	0.600 (0.486-0.714) p < 0.05	0.507 (0.303-0.711) p < 0.05	0.658 (0.581-0.735) p < 0.05
SynthEHR-Eviction (biobert-v1.1)	0.863 (0.851-0.874) p < 0.05	0.623 (0.478-0.767) p < 0.05	0.564 (0.418-0.711) p < 0.05	0.683 (0.611-0.756) p < 0.05
SynthEHR-Eviction (Bio_ClinicalBERT)	0.857 (0.847-0.867) p < 0.05	0.646 (0.601-0.692) p < 0.05	0.423 (0.395-0.451) p < 0.05	0.642 (0.631-0.654) p < 0.05
SynthEHR-Eviction (LLama-3.1-8B-FT)	0.968(0.962−0.973)_p<0.001	0.847 (0.821-0.874)	0.833 (0.798-0.868)	0.883 (0.866-0.900)
SynthEHR-Eviction (LLama-3.2-3B-FT)	0.914 (0.840-0.988)	0.642 (0.559-0.725) p < 0.05	0.782 (0.723-0.841) p < 0.05	0.780 (0.736-0.824) p < 0.05
SynthEHR-Eviction (Qwen2.5-7B-FT)	0.965 (0.958-0.973) p < 0.001	0.864(0.852−0.877)_	0.879(0.835−0.923)_	0.903(0.888−0.918)_p<0.05
SynthEHR-Eviction (Qwen2.5-3B-FT)	0.950 (0.942-0.958) p < 0.001	0.727 (0.646-0.808) p < 0.05	0.785 (0.749-0.821) p < 0.05	0.821 (0.787-0.855) p < 0.05
GPT-4o-APO (baseline)	0.900 (0.877-0.923)	0.850 (0.837-0.863)	0.870 (0.827-0.913)	0.873 (0.852-0.894)
GPT-4o-mini	0.836 (0.830-0.842) p < 0.05	0.703 (0.698-0.708) p < 0.05	0.685 (0.669-0.701) p < 0.05	0.741 (0.734-0.748) p < 0.05
GPT-4o-mini-APO	0.923 (0.904-0.942)	0.783 (0.725-0.840) p < 0.05	0.852 (0.806-0.898)	0.852 (0.827-0.878) p < 0.05
GPT-4o	0.858 (0.851-0.866) p < 0.05	0.851 (0.839-0.864) p < 0.05	0.804 (0.791-0.817) p < 0.05	0.838 (0.831-0.844) p < 0.05
SynthEHR-Eviction (bert_base_cased)	0.965 (0.958-0.973) p < 0.01	0.600 (0.486-0.714) p < 0.05	0.589 (0.415-0.763) p < 0.05	0.718 (0.621-0.815) p < 0.05
SynthEHR-Eviction (biobert-v1.1)	0.963 (0.951-0.974) p < 0.01	0.623 (0.478-0.767) p < 0.05	0.604 (0.443-0.765) p < 0.05	0.730 (0.627-0.833) p < 0.05
SynthEHR-Eviction (Bio_ClinicalBERT)	0.961 (0.956-0.966) p < 0.01	0.630 (0.612-0.648) p < 0.05	0.478 (0.459-0.497) p < 0.05	0.690 (0.683-0.696) p < 0.05
SynthEHR-Eviction (LLama-3.1-8B-FT)	**0.968(0.963−0.973)_ p** **<** **0.01**	0.850(0.820-0.880)	**0.926(0.910−0.942)_p<0.05**	**0.915(0.901−0.929)_**
SynthEHR-Eviction (LLama-3.2-3B-FT)	0.939 (0.917-0.960)	0.817 (0.787-0.847) p < 0.05	0.874 (0.844-0.904)	0.876 (0.856-0.897)
SynthEHR-Eviction (Qwen2.5-7B-FT)	0.965 (0.958-0.973) p < 0.01	0.866 (0.854-0.877)	0.907 (0.871-0.944)	0.913 (0.900-0.925) p < 0.05
SynthEHR-Eviction (Qwen2.5-3B-FT)	0.951 (0.944-0.959) p < 0.01	0.800 (0.769-0.831) p < 0.05	0.837 (0.818-0.856) p < 0.05	0.863 (0.849-0.877) p < 0.05
GPT-4o-APO (baseline)	0.910(0.877-0.943)	**0.878(0.853−0.904)**	0.878(0.833-0.922)	0.889(0.867-0.911)

We report both Macro-F1 and Micro-F1 scores with 95% confidence intervals for Step 1 (Binary Classification), Step 2 (Eviction Multi-Class Classification), and Step 3 (Non-Eviction Multi-Class Classification). *SynthEHR-Eviction* refers to models fine-tuned using the SynthEHR-Eviction dataset created via our Augmenter + Annotator pipeline. *GPT-4o-APO* serves as the baseline for statistical comparisons in each subtask. Blue cells indicate scores that are significantly lower than the baseline, orange cells indicate scores that are not significantly different from the baseline (i.e., at a similar level as GPT-4o-APO), red cells indicate scores that are significantly higher than the baseline, and represent **bold under lined values** the best performance within each column (i.e., the highest Micro-F1 or Macro-F1 for that dataset/subtask).

Open-source LLMs fine-tuned on the SynthEHR-Eviction dataset also demonstrated strong performance. Qwen2.5-7B-FT maintained robust results across all datasets (Synth: 0.963, MIMIC: 0.912, PMC: 0.891), achieving an average F1 of 0.922. LLaMA-3.1-8B-FT performed similarly well, with an average score of 0.908, and achieved particularly strong results on the Synth dataset (0.966). Although 3B-scale models (e.g., LLaMA-3.2-3B-FT, Qwen2.5-3B-FT) performed competitively on Synth, they lagged behind on PMC, indicating that smaller models may struggle with longer or more complex clinical texts.

BERT-based models fine-tuned with our SynthEHR-Eviction pipeline also yielded strong results on the Synth dataset. Notably, bert_base_cased (0.976), biobert-v1.1 (0.987), and Bio_ClinicalBERT (0.978) all outperformed GPT-4o-APO on this dataset. While their performance was less consistent on MIMIC (ranging from 0.756 to 0.916) and generally lower than GPT-4o-APO on PMC, their overall averages remained competitive. For instance, biobert-v1.1 achieved an average F1 of 0.931, slightly surpassing Qwen2.5-7B-FT. This strong performance is likely due to the relative simplicity of the binary classification task and the domain alignment of these biomedical encoders, which are well-suited for short-span eviction cue detection.

### Step 2: main results for multi-class classification of eviction subcategories

Eviction multi-class classification is substantially more challenging than binary classification because it requires distinguishing between seven fine-grained eviction subcategories (i.e., eviction-specific categories defined in Supplementary Table S6). In this more challenging setup, two strategies demonstrated clear value: fine-tuning with the SynthEHR-Eviction dataset and DSPy-based automated prompt optimization.

First, APO substantially improved GPT-4o and GPT-4o-mini. For GPT-4o, Macro-F1 increased from 0.515 to 0.878 and Micro-F1 from 0.613 to 0.886. For GPT-4o-mini, Macro-F1 rose from 0.273 to 0.691 and Micro-F1 from 0.433 to 0.764. These improvements mirror the APO trends observed in Step 1.

Meanwhile, models fine-tuned on SynthEHR-Eviction achieved competitive and often stronger performance than the GPT-4o-APO baseline. Qwen2.5-7B-FT reached a Macro-F1 of 0.920 and a Micro-F1 of 0.921 on the Synth dataset, slightly surpassing GPT-4o-APO’s 0.918 and 0.909. On MIMIC, it scored 0.939 (Macro-F1) and 0.938 (Micro-F1), again marginally higher than GPT-4o-APO’s 0.878 and 0.883. On PMC, Qwen2.5-7B-FT’s performance was more comparable, with a Macro-F1 of 0.804 vs. 0.836 and Micro-F1 of 0.824 vs. 0.868. Averaged across datasets, Qwen2.5-7B-FT achieved 0.888 (Macro-F1) and 0.895 (Micro-F1), slightly exceeding GPT-4o-APO’s 0.878 and 0.886. LLaMA-3.1-8B-FT also performed robustly, with Macro-F1/Micro-F1 scores of 0.881/0.906 (Synth), 0.935/0.925 (MIMIC), and 0.848/0.861 (PMC), leading to averages of 0.888 and 0.897, again on par with or better than GPT-4o-APO. Importantly, 3B-scale models such as LLaMA-3.2-3B-FT and Qwen2.5-3B-FT also performed competitively, suggesting that SynthEHR-Eviction enables scalable fine-tuning across model sizes. These 3B models achieved average Micro-F1 scores of 0.871 and 0.874, respectively, demonstrating their deployment potential in resource-constrained environments without sacrificing much performance.

In contrast, BERT-based biomedical models like BioBERT and Bio_ClinicalBERT struggled to generalize across datasets. Their average Macro-F1 and Micro-F1 scores remained below 0.70 and 0.72, respectively, with especially poor results on PMC. This suggests that while these models may encode biomedical terminology, they lack the representational flexibility needed for nuanced, fine-grained classification tasks like eviction status detection.

### Step 3: main results for multi-class classification of non-eviction subcategories

Unlike the eviction-specific classification in Step 2, the Step 3 task covers a broader set of non-eviction SDoH categories (social risk categories), including homelessness, housing instability, food insecurity, transportation insecurity, and others (see Supplementary Table S7). Many of these categories are commonly documented in public health and clinical records, making them more familiar to general-purpose LLMs. This is reflected in the relatively strong performance of GPT-4o even without APO, which achieved average scores of 0.797 Macro-F1 and 0.838 Micro-F1, significantly higher than its performance in Step 2.

Nonetheless, APO improved closed-source models, yielding performance gains consistent with those observed in earlier steps. These results, aligned with Steps 1–2, highlight APO’s broad utility across classification tasks.

Fine-tuned models based on SynthEHR-Eviction also demonstrated excellent performance. Most notably, Qwen2.5-7B-FT achieved a Macro-F1 of 0.903 and a Micro-F1 of 0.913, both significantly outperforming the GPT-4o-APO baseline (p < 0.05). LLaMA-3.1-8B-FT matched this strong performance with 0.883 and 0.915, respectively. These results highlight that even when tasks are not strictly “cold-start,” fine-tuning with task-specific high-quality data remains valuable for enhancing performance.

Encouragingly, 3B-scale models also performed well. Qwen2.5-3B-FT achieved 0.821 (Macro-F1) and 0.863 (Micro-F1), while LLaMA-3.2-3B-FT followed with 0.780 and 0.876, respectively, both clearly surpassing GPT-4o-mini-APO. These results demonstrate that SynthEHR-Eviction facilitates efficient scaling, enabling smaller models to reach production-quality performance without reliance on massive human annotation resources.

Finally, the biomedical BERT variants again underperformed across metrics (Macro-F1 < 0.69, Micro-F1 < 0.74), with particularly weak results on MIMIC and PMC. These models may lack the generative flexibility and contextual reasoning needed for multi-class prediction across heterogeneous SDoH categories.

In summary, while Step 3 categories are more likely to have been seen during LLM pretraining, SynthEHR-Eviction still offers substantial fine-tuning benefits. When combined with its superior performance on unseen (eviction-specific) categories, this suggests that our solution serves as a robust and broadly applicable resource for improving SDoH modeling in both known and novel scenarios.

### Class performance variations across eviction categories

Table 2 reports the class-wise performance of five models (e.g., GPT-4o-APO, Llama-3.1-8B-FT, Llama-3.2-3B-FT, Qwen2.5-7B-FT, and Qwen2.5-3B-FT) on seven nuanced eviction-related categories across three datasets (Synth, Mimic, PMC). These classes vary in eviction status (e.g., present, pending, hypothetical), legal framing (e.g., mutual rescission), and temporal cues (e.g., current vs. history).

**Table 2:** Model performance comparison across different datasets (Synth, Mimic, PMC) with MCC and F1 scores (including Macro-F1 and Micro-F1 in Overall) for each eviction class

		Synth		Mimic		PMC		Avg	
Model	Class	MCC	F1	MCC	F1	MCC	F1	MCC	F1
GPT-4o-APO	Eviction_absent	1.00	1.00					1.00	1.00
	Eviction_hypothetical	1.00	1.00	0.75	0.75	0.83	0.84	0.86	0.86
	Eviction_mr_current	0.82	0.84	0.89	0.90	0.95	0.96	0.89	0.90
	Eviction_mr_history	0.81	0.81	0.88	0.89	0.89	0.89	0.86	0.86
	Eviction_pending	0.95	0.96	0.81	0.83	0.81	0.83	0.86	0.87
	Eviction_present_current	0.91	0.93	0.97	0.97	0.88	0.89	0.92	0.93
	Eviction_present_history	0.94	0.94	0.97	0.98	0.95	0.96	0.95	0.96
	Overall_(macro_f1/micro_f1)	0.92	0.93/0.93	0.88	0.89/0.89	0.88	0.89/0.90	0.89	0.90/0.91
Llama-3.1-8B-FT	Eviction_absent	1.00	1.00					1.00	1.00
	Eviction_hypothetical	0.97	0.98	0.94	0.95	0.91	0.92	0.94	0.95
	Eviction_mr_current	0.77	0.80	0.94	0.95	0.89	0.90	0.87	0.88
	Eviction_mr_history	0.87	0.89	0.94	0.95	0.76	0.80	0.86	0.88
	Eviction_pending	0.87	0.88	0.94	0.95	0.81	0.83	0.87	0.89
	Eviction_present_current	0.88	0.90	1.00	1.00	0.74	0.76	0.88	0.89
	Eviction_present_history	0.91	0.92	1.00	1.00	0.85	0.87	0.92	0.93
	Overall_(macro_f1/micro_f1)	0.89	0.80/0.91	0.96	0.97/0.97	0.82	0.85/0.85	0.89	0.87/0.91
Llama-3.2-3B-FT	Eviction_absent	1.00	1.00					1.00	1.00
	Eviction_hypothetical	1.00	1.00	0.94	0.95	0.94	0.95	0.96	0.97
	Eviction_mr_current	0.81	0.83	0.90	0.92	0.88	0.89	0.86	0.88
	Eviction_mr_history	0.87	0.89	0.92	0.93	0.79	0.82	0.86	0.88
	Eviction_pending	0.89	0.91	0.92	0.93	0.83	0.85	0.88	0.90
	Eviction_present_current	0.89	0.89	0.80	0.81	0.75	0.76	0.81	0.82
	Eviction_present_history	0.91	0.92	0.92	0.93	0.89	0.91	0.91	0.92
	Overall (macro_f1/micro_f1)	0.91	0.92/0.92	0.90	0.91/0.92	0.84	0.86/0.87	0.89	0.90/0.90
Qwen2.5-7B-FT	Eviction_absent	1.00	1.00					1.00	1.00
	Eviction_hypothetical	0.97	0.98	0.91	0.92	0.88	0.89	0.92	0.93
	Eviction_mr_current	0.87	0.88	0.94	0.95	0.94	0.95	0.91	0.93
	Eviction_mr_history	0.94	0.94	0.94	0.95	0.85	0.87	0.91	0.92
	Eviction_pending	0.89	0.91	0.87	0.89	0.77	0.80	0.85	0.87
	Eviction_present_current	0.91	0.93	0.94	0.94	0.71	0.73	0.85	0.87
	Eviction_present_history	0.91	0.92	1.00	1.00	0.88	0.90	0.93	0.94
	Overall (macro_f1/micro_f1)	0.93	0.94/0.94	0.93	0.94/0.94	0.84	0.86/0.86	0.90	0.91/0.91
Qwen2.5-3B-FT	Eviction_absent	1.00	1.00					1.00	1.00
	Eviction_hypothetical	0.91	0.93	0.81	0.82	0.88	0.89	0.87	0.88
	Eviction_mr_current	0.84	0.86	0.87	0.89	0.92	0.93	0.88	0.89
	Eviction_mr_history	0.87	0.88	0.88	0.90	0.85	0.86	0.87	0.88
	Eviction_pending	0.87	0.89	0.79	0.82	0.77	0.80	0.81	0.84
	Eviction_present_current	0.91	0.93	0.84	0.85	0.78	0.79	0.84	0.85
	Eviction_present_history	0.91	0.92	0.95	0.95	0.92	0.93	0.92	0.93
	Overall (macro_f1/micro_f1)	0.90	0.91/0.91	0.86	0.87/0.88	0.85	0.87/0.87	0.87	0.88 /0.89

Results present four models: GPT-4o-APO, Llama-3.1-8B-FT, Llama-3.2-3B-FT, Qwen2.5-7B-FT, and Qwen2.5-3B-FT, with overall average metrics. Class counts are fixed within each dataset and identical across all evaluated models. Specifically, for eviction-related classes, Synth and MIMIC each contain 20 samples per class, while PMC contains 8 samples per class. Detailed dataset statistics and class distributions are reported in Table 3. Note that “Eviction_absent" appears exclusively in the Synth dataset as it functions as a critical negative class that establishes decision boundaries between eviction-relevant and irrelevant content during model training. Mimic and PMC datasets deliberately omit this class to focus evaluation specifically on discrimination capabilities between different positive eviction scenarios. Importantly, this omission is not due to data scarcity. On the contrary, most real-world clinical notes in MIMIC and PMC would fall under the “Eviction_absent" category. However, including such easy negatives would inflate overall accuracy and obscure the model’s ability to distinguish among nuanced positive classes, so they were excluded by design.

All models achieved perfect classification (F1 = 1.00) on Eviction_absent in the Synthetic dataset. This class was excluded from Mimic and PMC to prevent dominance of trivial negatives and to emphasize the model’s ability to differentiate among positive cases.

Eviction_hypothetical, representing projected or threatened eviction, posed moderate challenges. GPT-4o-APO reached an average F1 of 0.86, while several fine-tuned open-source LLMs, specifically Qwen2.5-7B-FT and Llama-3.2-3B-FT, surpassed this benchmark with Synth scores of 0.98 and 1.00, and solid generalization in Mimic (0.92 and 0.95), highlighting the benefit of domain-specific fine-tuning on SynthEHR-Eviction.

Eviction_mr_current and Eviction_mr_history, which capture mutually agreed lease terminations, were consistently well-handled. Qwen2.5-7B-FT achieved 0.93 and 0.92 on Macro-F1 and Micro-F1, respectively, slightly outperforming GPT-4o-APO (F1 = 0.90 and 0.86). Notably, fine-tuned 3B models also maintained high accuracy in these categories (F1 is about 0.88), suggesting strong transfer of structured legal signals even in smaller models after fine-tuning.

Eviction_pending, indicating formal but unresolved eviction processes, remained somewhat ambiguous. Qwen2.5-7B-FT and Llama-3.1-8B-FT achieved F1 = 0.91 and 0.95 in Synth and Mimic, respectively, surpassing GPT-4o-APO’s average of 0.87. However, smaller models showed more variance across datasets, especially in PMC.

**Fig. 1:**
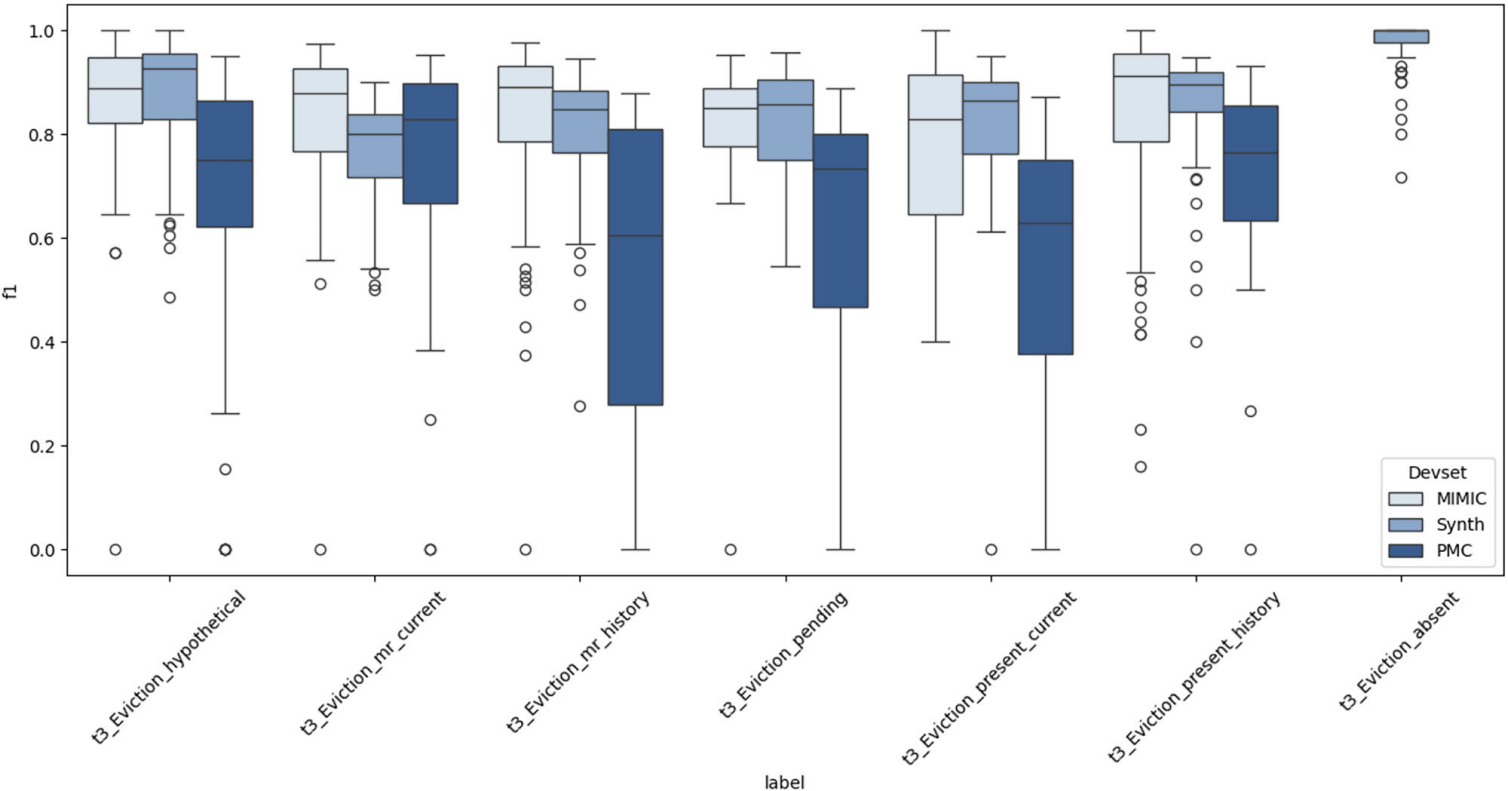
Class-wise performance distributions for Step 2 (Eviction Multi-Class) across Synth, MIMIC, and PMC datasets. Box plots show F1 score variability across models, revealing pronounced performance differences between temporally ambiguous categories (e.g., present_current vs. present_history), particularly under domain shift in PMC notes.

Eviction_present_current and Eviction_present_history, which rely on subtle differences in recency and documentation of completed evictions, remained the most challenging. In PMC, for example, Llama-3.1-8B-FT dropped to 0.76 on “present_current", while GPT-4o-APO maintained a stronger F1 of 0.93. These results highlight that while fine-tuned LLMs can excel in categories grounded in legal constructs, temporal ambiguity still presents difficulties. Figure 1 further visualizes these class-level differences by showing the distribution of F1 scores across datasets, highlighting systematic performance gaps between current and history eviction categories.

In summary, class-level comparisons reveal that fine-tuned open-source LLMs, especially Qwen2.5-7B-FT and Llama-3.2-3B-FT, can match or exceed GPT-4o-APO in multiple eviction categories after training on SynthEHR-Eviction. Larger models (7B/8B) showed better generalization across datasets, while smaller models (3B) still offered strong performance on well-defined categories. GPT-4o-APO provided a competitive baseline with consistent performance across the board, though not uniformly superior to well-trained open models.

### Impact of reasoning in training data

**Fig. 2:**
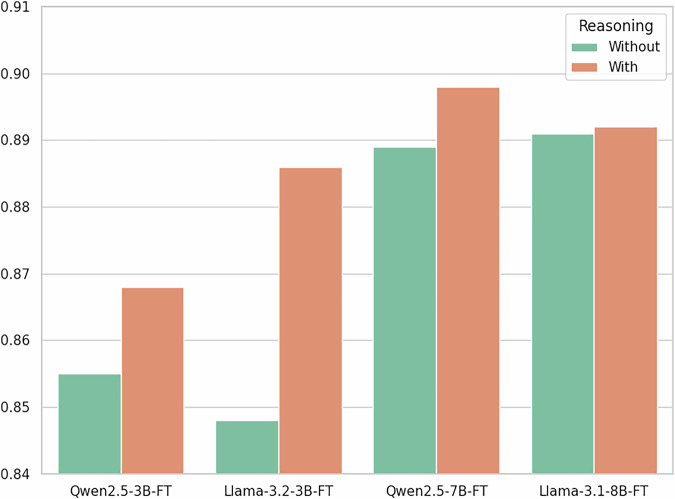
Performance comparison of LLMs trained with and without reasoning annotations on the Eviction Multi-Class task.

To evaluate the effect of explicit reasoning annotations in training data, we prepared two versions of each model’s training set: one with reasoning explanations and one without, keeping the labels identical. As shown in Fig. 2, smaller models benefit more noticeably from reasoning annotations. For instance, Llama-3.2-3B-FT improved from 0.848 to 0.886 (+3.8), and Qwen2.5-3B-FT improved from 0.855 to 0.868 (+1.3). In contrast, larger models like Qwen2.5-7B-FT and Llama-3.1-8B-FT maintained stable performance with or without reasoning, with minimal differences (less than 0.01). These findings suggest that the reasoning annotations generated through the SynthEHR-Eviction pipeline can provide varying degrees of structured guidance, especially for smaller models that benefit from clearer decision logic in complex classification tasks. Additionally, models trained with these annotations are capable of generating their own reasoning during inference, improving transparency and interpretability. This makes them more amenable to expert review and downstream validation, moving beyond black-box eviciton prediction. We note that all models were trained under identical task-specific optimization budgets; additional details on training steps and sequence-length effects are provided in the Supplementary Information (Fine-tuning Settings).

### Training data size impact on finetuning

To examine how training data size affects model performance, we conducted systematic experiments on two traditional BERT-based models (BERT and BioBERT) and four open-source LLMs (two 3B-scale and two 7B/8B-scale LLMs). We evaluated both the Eviction Multi-Class and Non-Eviction Multi-Class tasks using training datasets of varying sizes (200 to 10,000 samples) and reported both Macro-F1 and Micro-F1 scores (Supplementary Figure S2).

Across both tasks, models demonstrated substantial gains when the training data was increased from 200 to 3000 samples. For instance, Qwen2.5-7B-FT improved its Macro-F1 on the Eviction task from 0.748 to 0.853, and on the Non-Eviction task from 0.75 to 0.891. Similarly, LLaMA-3.1-8B-FT increased from 0.670 to 0.857 (Eviction) and from 0.736 to 0.893 (Non-Eviction). Performance gains typically plateaued beyond 5000 samples, and several models exhibited signs of overfitting at 10,000 samples. For example, Qwen2.5-7B-FT reached its peak Macro-F1 of 0.912 on the Eviction task at 5000 samples, then dropped to 0.850 at 10,000 samples.

Optimal dataset sizes varied by task. For Step 2 (Eviction), LLMs such as Qwen2.5-7B-FT required larger datasets to reach optimal performance (e.g., 0.912 Macro-F1 at 5000 samples). In Step 3 (Non-Eviction), most models converged earlier, reaching their peak performance around 3000 samples (e.g., Qwen2.5-7B-FT at 0.891).

For practical fine-tuning, training with 3000-5000 samples provides a strong balance between performance and efficiency, since several LLMs trained on just 3000-5000 samples reached performance levels comparable to GPT-4o-APO. Accordingly, we used 5000 samples for LLMs and 3000 samples for traditional BERT models in Step 2. For Step 3, we used 3000 samples for both model types, as performance had largely converged at this size Table 3.

**Table 3:** Data statistics across training and evaluation splits for APO (for close-source models) and SFT (for open-source models)

Label	DSPy-train (Synth)	DSPy-eval (Synth)	SFT-train+eval	Test-total	Test-Synth	Test-Mimic	Test-PMC
Eviction_absent	8	12	500	20	20		
Eviction_hypothetical	8	12	750	48	20	20	8
Eviction_mr_current	8	12	750	48	20	20	8
Eviction_mr_history	8	12	750	48	20	20	8
Eviction_pending	8	12	750	48	20	20	8
Eviction_present_current	8	12	750	48	20	20	8
Eviction_present_history	8	12	750	48	20	20	8
Eviction_total	56	84	5000	308	140	120	48
Homelessness	8	12	450	20	20		
InadequateHousing	8	12	450	48	20	20	8
LackOfAdequateFood	8	12	450	48	20	20	8
FinancialInsecurity	8	12	450	48	20	20	8
HousingInstability	8	12	450	48	20	20	8
MaterialHardship	8	12	450	48	20	20	8
TransportationInsecurity	8	12	300	48	20	20	8
Non-Eviction_total	56	84	3000	308	140	120	48

## Discussion

Eviction is a critical yet underrepresented SDoH, associated with cascading adverse outcomes such as homelessness, chronic stress, and emergency care overuse^13–15,17,27,32^. While prior NLP efforts have focused on broader housing-related issues, such as homelessness or housing instability^11,26^, the status of eviction has been largely neglected in public clinical datasets and benchmarks. Only very recently has this issue received direct attention. For instance, Yao et al. (2023) introduced an eviction classifier trained on a private Veterans Affairs dataset^27^. To help fill this gap, we present SynthEHR-Eviction: a scalable and clinically grounded data generation pipeline that produces high-fidelity, eviction-labeled clinical notes aligned with real-world documentation practices. The pipeline combines LLM-based data augmentation, automated prompt optimization, and human-in-the-loop validation to synthesize instruction-style notes paired with labels derived from ICD-10 Z59 codes (e.g., Z59.0-Z59.9, covering housing and economic instability). This label schema enhances semantic precision and interoperability with structured health data systems^11,20^. Furthermore, our taxonomy goes beyond binary classification by capturing nuanced eviction subtypes—such as pending status, mutual rescission, and temporal distinctions (e.g., current vs. history) This allows for more accurate model training and more actionable risk detection. By releasing both the annotated dataset and the labeling guidelines, we support the development and evaluation of eviction-aware NLP tools that generalize across institutions and reflect realistic variability in documentation.

One of the most practical contributions of SynthEHR-Eviction lies in its efficiency: it dramatically reduces the human effort required to construct large-scale, high-quality annotated datasets. Manual annotation of complex SDoH categories, particularly those like eviction that involve temporal ambiguity, legal framing, and implicit cues, is notoriously labor-intensive. In our controlled study, full manual rewriting of 8,000 EHR notes took over 266 hours, and even direct annotation of labels consumed 33 hours. In contrast, our GPT-assisted, human-in-the-loop workflow, with targeted prompt engineering, batch validation, and automated reasoning trace generation, achieved comparable data quality with less than 6 hours of human effort, representing an over 80% reduction in annotation time. These findings align with recent work showing the scalability and reliability of weak supervision, prompt-based augmentation, and LLM-powered data generation in other domains^33–36^. More importantly, our pipeline offers structured reasoning alongside each label, allowing smaller models to learn decision rationales explicitly—an advantage not typically found in weakly labeled or distant supervision setups. Because the pipeline is built on modular components (e.g., task-adaptive prompt optimization, reasoning-based CoT supervision, and human verification loops), it generalizes to other low-resource SDoH domains where fine-grained annotation is scarce but clinically significant (e.g., utility shutoff, food insecurity, or intimate partner violence). As large-scale clinical NLP increasingly shifts toward low-cost, explainable, and domain-adaptable solutions, SynthEHR-Eviction provides a blueprint for scaling up dataset construction without compromising semantic depth or real-world alignment^11,30^.

To systematically evaluate model generalization in eviction-related SDoH classification, we tested performance across three distinct clinical datasets, Synth, MIMIC, and PMC, that vary substantially in linguistic style, narrative density, and documentation patterns (Supplementary Table S9). Our results reveal a consistent domain shift: models achieved their highest performance on the synthetic data (Synth), performed moderately on real-world EHRs (MIMIC), and performed worst on academic case reports (PMC). While all three datasets adopt instruction-style formatting, they differ in vocabulary, structure, and contextual framing of eviction signals. The Synth dataset, generated using our LLM-based pipeline, closely mirrors the model’s generation style and served as the primary training source, explaining its strong in-domain performance. In contrast, MIMIC notes, written by clinicians, feature more heterogeneous phrasing (e.g., “forced to remove from the rented house”) and exhibit subtle lexical shifts. The PMC notes pose the greatest challenge, as eviction information is often deeply embedded in long diagnostic narratives and may appear only incidentally in unrelated sections, requiring fine-grained relevance detection and temporal disambiguation (as shown in Supplementary Table S9). This distributional mismatch explains the observed drop in generalization: for example, Qwen2.5-7B-FT’s Macro-F1 fell from 0.920 (Synth) to 0.804 (PMC). These findings are consistent with prior SDoH extraction studies documenting performance degradation when models are evaluated on free-text narratives^25,37,38^. Notably, a simple data composition intervention, replacing just 30% of synthetic training data with real PMC notes, led to substantial gains in out-of-domain performance. On the PMC portion of the Step 2 task, Qwen2.5-7B-FT’s Micro-F1 improved by +0.302 (Supplementary Table S1). This result reinforces a growing consensus in clinical NLP: while synthetic data offers scale and control, it is insufficient on its own for achieving robust generalization. Incorporating even a small proportion of real-world examples into training is essential for building models that transfer reliably to naturalistic clinical settings^11^. Moreover, because SynthEHR-Eviction spans synthetic and real-world document styles, it enabled us to evaluate how different model families generalize across varied clinical documentation settings. On the challenging PMC multi-class classification task, a general-domain LLaMA-3.1-8B-FT model achieved a Micro-F1 of 0.861, while the BERT-base-cased model fell to just 0.444, a difference of over 40 percentage points. This substantial gap underscores LLMs’ superior ability to handle diverse and noisy clinical narratives, likely due to their larger parameter counts, broader pretraining corpora, and emergent reasoning capabilities^11^. These findings are in line with recent benchmarks showing that state-of-the-art LLMs, such as GPT-4 and Flan-T5-XXL, consistently outperform domain-specific biomedical models like BioBERT and ClinicalBERT across SDoH extraction and other fine-grained clinical NLP tasks^11,39^. However, we also recognize that LLM deployment in real-world settings presents nontrivial challenges: inference latency, compute costs, and limited GPU infrastructure may hinder adoption in under-resourced health systems^40^. Thus, future work should explore hybrid strategies such as model distillation, dynamic inference control, or task-specific adapter modules to achieve a more practical balance between performance and feasibility. A nuanced understanding of this tradeoff is critical to ensuring that SDoH-aware NLP tools are not only accurate but also equitable and deployable at scale.

Our fine-grained eviction label schema, distinguishing categories such as Eviction_present_current and Eviction_present_history, allowed us to examine one of the most persistent challenges in clinical NLP: temporal reasoning errors arising from implicit event timelines in free-text notes. Temporal ambiguity emerged as a consistent source of classification error during model evaluation. A closer inspection of eviction status classification errors (Cases 1 and 2, Supplementary Table S12) reveals a critical challenge: the models’ difficulty in distinguishing between historical and current eviction events due to implicit temporal ambiguities in clinical notes. In Case 1, the phrase “resides now" prompted the Llama-3.1-8B-FT model to incorrectly classify a historical eviction (Eviction_present_history) as ongoing (Eviction_present_current). Conversely, in Case 2, the model incorrectly labeled a recent eviction ("two months ago") as historical, heavily influenced by the initial lexical cue “history," despite the explicit recent temporal context. These bidirectional error patterns emphasize temporal ambiguity, where historical events can have ongoing impacts or be conflated with current circumstances. Notably, our observations align with known challenges in clinical NLP: accurately capturing the timing of events in narratives is difficult for models^41^. Even advanced LLMs have been reported to struggle with temporal relation extraction, performing worse than fine-tuned models and showing inconsistent handling of event order. This persistent ambiguity has been documented since earlier efforts, such as THYME^42^ and the Clinical TempEval challenges^43^, and remains a focus of current research. Our findings reinforce that temporal context (e.g., distinguishing a resolved eviction in the past from a patient’s current housing crisis) is a critical source of error. To further investigate and mitigate temporal ambiguity, we conducted an additional experiment that explicitly decouples temporal reasoning from eviction status classification. Rather than jointly predicting temporally entangled labels, we focus on four eviction-related classes that involve temporal distinctions and introduce a second-stage classifier that predicts *current* versus *history* based on the clinical note and the predicted eviction status. This task decomposition substantially improves temporal classification performance. Fine-tuning Qwen-2.5-7B for this second-stage task increases Macro-F1 from 0.50 to 0.91 and MCC from 0.2881 to 0.82, approaching GPT-4o. These results suggest that separating temporal classification from eviction status prediction can effectively mitigate temporal ambiguity in clinical notes. Addressing these temporal reasoning issues is vital, as accurately determining whether an eviction is ongoing or historical is crucial for effective risk assessment and intervention.

We also explored the use of structured reasoning annotations, i.e., chain-of-thought explanations provided during training, to examine their utility in enhancing model performance and interpretability, particularly for smaller, resource-efficient models. Our experiments showed that smaller models benefit disproportionately from this form of supervision: LLaMA-3.2-3B-FT improved by +3.8 Macro-F1 and Qwen2.5-3B-FT by +1.3, compared to their counterparts trained on labels alone. In contrast, larger models (Llama-3.1-8B-FT, Qwen2.5-7B-FT) showed minimal improvements under the same setup, indicating that extensive pretraining had already endowed them with strong inherent reasoning abilities. These findings are consistent with prior observations on chain-of-thought prompting^44^: smaller models often struggle to exhibit complex reasoning capabilities unless explicitly guided, while larger models tend to benefit less from such supervision. SynthEHR-Eviction operationalizes this insight by pairing each label with interpretable reasoning traces, allowing smaller models to learn not only what the correct answer is but also why it is correct. Our results reinforce this trend, demonstrating that reasoning-guided training can substantially improve the performance of smaller models, while offering limited additional value for larger ones. This performance disparity helps explain why our 3B-scale models required explicit reasoning supervision to accurately distinguish fine-grained eviction categories, while the 7-8B models performed comparably well without it. Qualitative analysis further supports this conclusion. In Case 3 (Supplementary Table S12), the Llama-3.2-3B-FT model trained with reasoning annotations correctly classified a complex eviction situation as Eviction_pending, thereby clearly articulating the eviction process’s active and unresolved nature. In contrast, the same model trained without reasoning annotations misclassified the scenario as Eviction_present_current, failing to capture the subtle temporal and procedural cues. This example illustrates how structured reasoning guidance can help smaller models develop more precise and interpretable decision boundaries in semantically challenging cases. From a deployment perspective, these results highlight the practical value of reasoning annotations in improving both the performance and interpretability of smaller, more resource-efficient models^45^. By leveraging the reasoning-enhanced supervision provided by SynthEHR-Eviction, smaller models can match or exceed the performance of much larger LLMs while remaining compatible with edge deployments and low-resource clinical infrastructure. This is particularly relevant in real-world clinical environments where compute budgets are constrained, such as community hospitals, mobile health units, or edge devices operating in under-resourced settings^40^. Reasoning-augmented training, as implemented in our pipeline, thus enables scalable and equitable NLP solutions for SDoH extraction.

While structured reasoning annotations improved model transparency, our evaluation further revealed limitations in reasoning fidelity, that is, whether a model’s explanation faithfully reflects its underlying decision process. Our case studies (Cases 4-6, Supplementary Table S13) revealed interpretability challenges even when the final classification was correct. In several instances, the model produced an explanation that was partially incorrect or conceptually misaligned, despite outputting the right label. For example, the Llama-3.1-8B-FT model correctly labeled a case as Eviction_mr_current (mutual rescission current), but its explanation erroneously stated that the mutual lease rescission was unrelated to eviction, indicating a misunderstanding of the concept (Case 4). In another case (Case 5), the model correctly identified the eviction as Eviction_pending but described it using language that suggests a historical context ("so we will label it as ‘history”’), which deviates from the annotation guidelines. Similarly, a note properly labeled as Eviction_present_current was explained as if the eviction process were still ongoing, failing to recognize it had been completed. These examples demonstrate that the presence of a reasoning chain does not guarantee its fidelity, that is, alignment with the model’s actual decision boundary or reasoning strategy. Recent studies^46,47^ on LLM explainability have similarly noted that models often generate plausible-sounding rationales that are not fully faithful to their internal computations, or even inconsistent with their final outputs. In other words, an AI can be “right for the wrong reasons,”^48–50^ as seen in our case analyses. This reinforces the need for rigorous human oversight and domain-informed evaluation when using reasoning annotations for interpretability. SynthEHR-Eviction provides such a platform, where both correctness and explanation quality can be jointly assessed. Our findings echo growing efforts to measure and improve the faithfulness of chain-of-thought explanations in LLMs^51^. Ensuring that the model’s explanations are not only present but also correct and useful is an important direction for future work, especially in high-stakes clinical applications^52^. This aligns with broader efforts in clinical NLP to reduce hallucinations and improve factual alignment in model-generated outputs^53–56^. Continual refinement of the reasoning templates and collaboration with clinical experts will be needed to close these gaps^49,50^.

Finally, our experiments suggest that model performance tends to level off between 3000 and 5000 training examples, with little improvement, and in some cases, slight overfitting, when more data is added. This threshold may reflect either the high informational density of our pipeline-generated data, where critical semantic cues are well-covered early, or diminishing marginal returns from increasingly repetitive synthetic notes. These hypotheses warrant further investigation. Nonetheless, our results suggest that 3000-5000 examples may offer a practical trade-off between data curation effort and fine-tuning performance for this task.

While our results demonstrate the promise of SynthEHR-Eviction, several limitations remain. First, although grounded in real EHR patterns, the synthetic training data may not capture the full variability and sociolinguistic nuance found in actual clinical documentation across all institutions and populations. This raises potential concerns about distributional bias and overfitting to synthetic patterns. Importantly, being a large public dataset does not imply comprehensive coverage of real-world linguistic variation: synthetic generation (and even MIMIC-style rewriting) cannot fully reproduce institution-specific abbreviations, templated note structures, and localized documentation practices. Importantly, these coverage limitations can also create equity risks: documentation of housing and legal stressors may differ across institutions and across patient populations, and synthetic generation may inadvertently reproduce biased or stereotyped socioeconomic cues. As a result, model errors may be unevenly distributed across groups or settings, which is especially concerning for downstream uses that could affect access to social services. Future work should prioritize multi-institutional validation and, where permitted by data governance, subgroup-aware auditing and clinician/social worker oversight to ensure eviction detection is used to support (not penalize) vulnerable patients. Second, we did not exhaustively explore all methods for generating synthetic data. We focused on prompt optimization and high-precision, reproducible, low-cost augmentation strategies, which may exclude higher-recall alternatives. Third, although the initial results are promising, additional clinical validation will be necessary to establish the model’s performance in real-world environments. Finally, as with any annotation effort, the quality of the output depends on the guidelines and decisions of human annotators. Even with structured reasoning supervision, SDoH annotation remains a complex task subject to ambiguity and inter-annotator variability. Future work should investigate how to balance synthetic and real data, address biases introduced by LLM pretraining, expand evaluation across multi-institution real-world EHR corpora, and assess the downstream utility of eviction detection in improving health outcomes and promoting equity.

Taken together, SynthEHR-Eviction provides a scalable and clinically grounded foundation for enhancing the detection of eviction-related SDoH in unstructured clinical notes. With its structured reasoning supervision, domain-diverse testbed, and interpretable outputs, the benchmark is particularly well-suited for evaluating and refining NLP models in socially sensitive, high-stakes settings. Beyond its methodological contributions, SynthEHR-Eviction introduces two broadly applicable design principles, domain-specific abstraction and task-aligned supervision, that can be extended to other underrepresented SDoH domains, such as legal hardship or caregiving burden. Its automated and modular pipeline supports low-cost construction of semantically rich datasets, enabling scalable integration of diverse social contexts into downstream clinical NLP applications. For example, when applied to AI hospital simulations, these SDoH-aware datasets can enrich the behavioral profiles of patient agents^57^, making model evaluations more holistic and socially grounded. By openly releasing our dataset, annotation schema, and reasoning templates, we further promote transparency, reproducibility, and community adoption. From a deployment perspective, the inclusion of structured reasoning outputs facilitates integration into real-world clinical workflows. For instance, coupling an “eviction risk” flag with a concise model-generated rationale could help social workers and care teams quickly interpret and act upon risk signals. With appropriate clinical validation, such NLP-based surveillance tools could be integrated into existing national infrastructures, such as the U.S. Veterans Affairs data ecosystem, where they would complement structured resources like the Homelessness Management Information System (HOMES) and enhance population-level dashboards such as USVETS, as demonstrated by our previous nationwide study on NLP-identified eviction rates among over 7 million veterans^58^. Other studies have shown that enriching structured fields with NLP-derived factors improves the identification of at-risk patients^11^. In this context, SynthEHR-Eviction could help operationalize timely referrals to social services, housing aid, or financial counseling, interventions that may avert escalation to homelessness or emergency medical crises. By enabling early and interpretable surfacing of eviction risk, SynthEHR-Eviction bridges a critical gap between clinical documentation and actionable care pathways. The structured rationales it produces not only improve transparency but also build clinician trust, two requirements for safe deployment in health systems. Looking forward, we envision this work as a stepping stone toward broader SDoH-focused informatics innovations, including fine-grained ICD-Z code modeling, hybrid symbolic-neural architectures for social reasoning, and equitable NLP systems tailored to population health. Ultimately, SynthEHR-Eviction demonstrates how modern LLM pipelines can be adapted to low-resource, high-impact clinical domains, advancing the broader agenda of embedding social medicine into the digital infrastructure of healthcare, a direction that is increasingly central to both health equity and precision public health.

## Methods

**Fig. 3:**
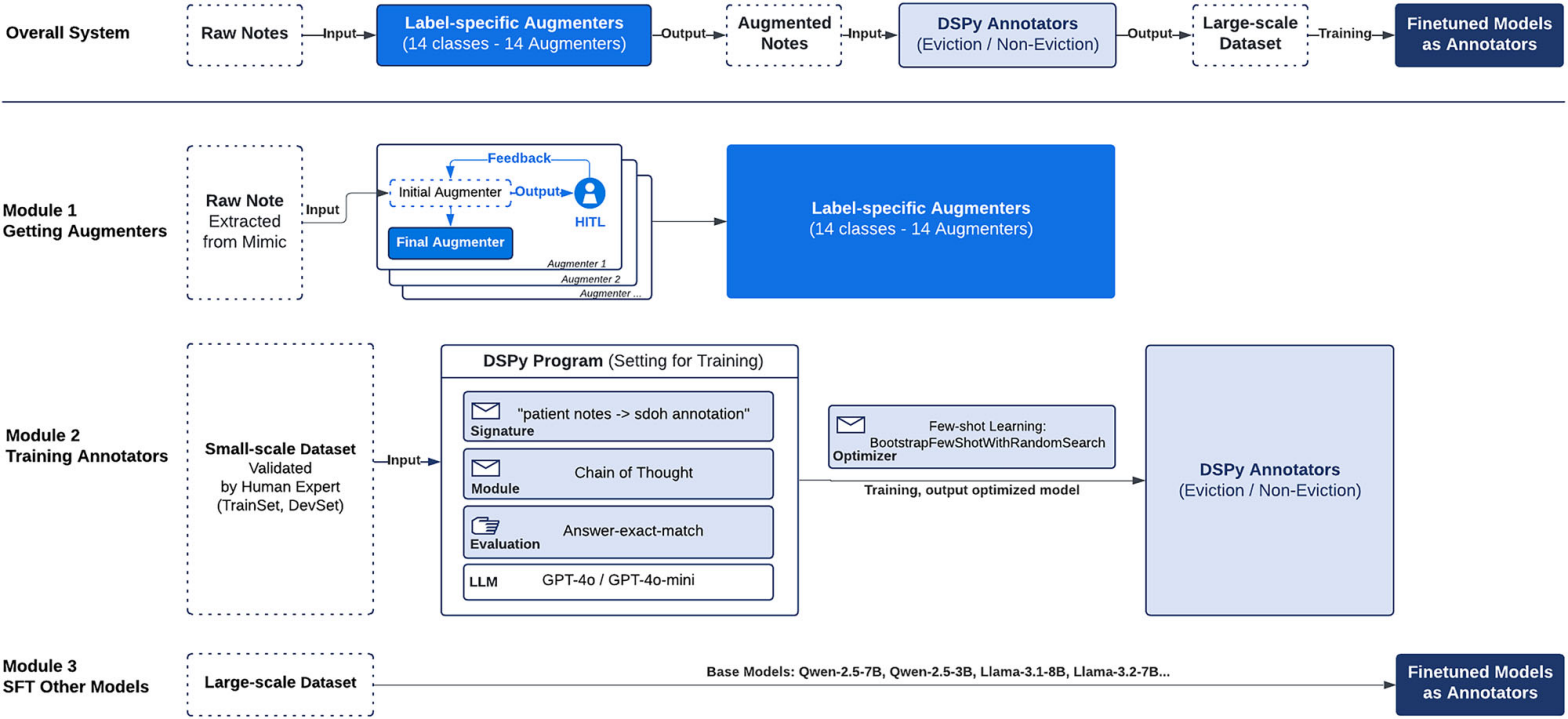
The pipeline consists of three modular stages. Overall System: Raw clinical notes are first processed by label-specific LLM augmenters (one augmenter per class) to generate augmented notes, which are then annotated by DSPy-optimized annotators to form a large-scale labeled dataset used for open-source model training. Module 1: Label-specific augmenters are obtained through an iterative human-in-the-loop process, where experts review initial augmenter outputs and provide feedback to refine the final augmenters. Module 2: A small expert-validated dataset is used to train DSPy annotators via automated prompt optimization, specifying task signatures, multi-step reasoning modules, and exact-match evaluation objectives. Module 3: The resulting large-scale synthetic and real dataset is used to fine-tune open-source language models, yielding scalable annotators for eviction and non-eviction classification. The note-level transformations illustrated in Supplementary Figure S1 exemplify the augmentation and annotation process applied throughout this pipeline.

As illustrated in Fig. 3, our approach focuses on developing a highly effective and reusable pipeline for multi-class annotation, which can be adapted to other SDoH annotation tasks. In this study, eviction serves as the primary experimental focus, showcasing the pipeline’s ability to generate high-quality annotations across multiple eviction-related categories. The system is divided into three primary components: a training process for DSPy, data augmentation pipeline, and annotation system. The training process utilizes a small-scale human-validated dataset to optimize the DSPy program; the data augmentation pipeline generates labeled samples from raw clinical records; and the annotation system applies the trained models to classify augmented data and provide reasoning.

### Data augmentation pipeline

**Fig. 4:**
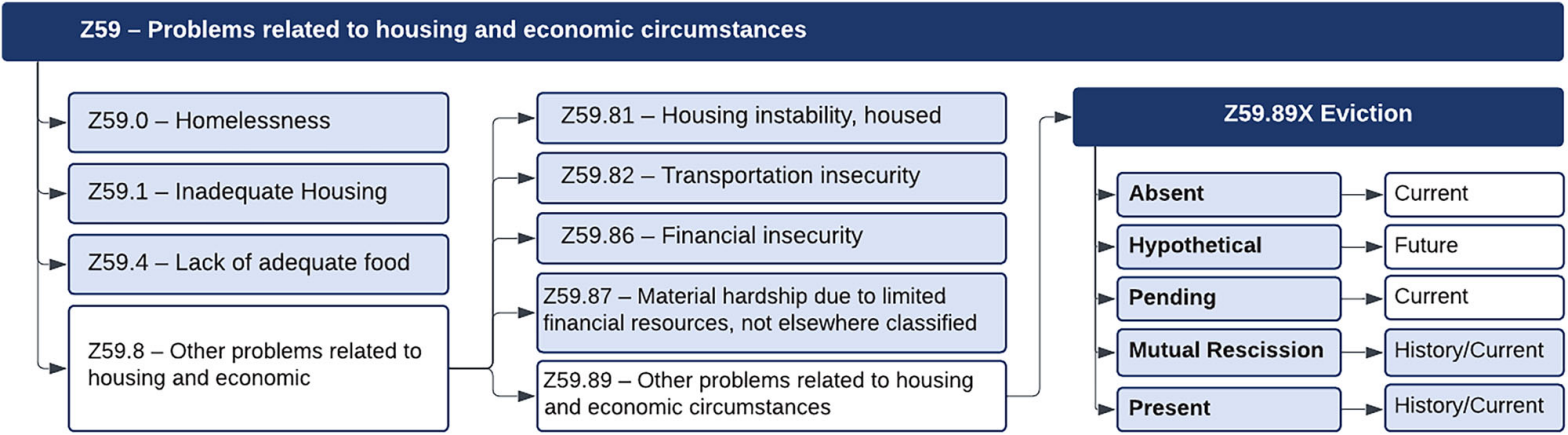
Classification framework for eviction and related housing and economic challenges. Colors highlight the classes included in our taxonomy: dark-grey boxes denote Tier 1 SDoH categories, light-grey boxes denote Tier 2 SDoH categories, and blue boxes denote eviction-related classes under Z59.89X. Tier 1 includes 3 categories: (1) Z59.0 Homelessness, (2) Z59.1 Inadequate housing, and (3) Z59.4 Lack of adequate food. Tier 2 includes 4 categories: (4) Z59.81 Housing instability, (5) Z59.82 Transportation insecurity, (6) Z59.86 Financial insecurity, and (7) Z59.87 Material hardship. Z59.89X defines 5 eviction-related presence states paired with their corresponding periods (Current, History): (8) Eviction Absent, (9) Eviction Hypothetical, (10) Eviction Pending, (11) Eviction Mutual Rescission-History, (12) Eviction Mutual Rescission-Current, (13) Eviction Present-History, and (14) Eviction Present-Current. A complete description and examples for all 14 classes are provided in Supplementary Tables S6 and S7.

First, we extracted raw notes by selecting over 30,000 discharge notes from MIMIC-III and MIMIC-IV and isolating the “social history” section as the augmentation input. This was based on our observation that most descriptions of patients’ social circumstances, such as housing, financial status, and other social determinants, were concentrated within this part of the discharge notes. We then extracted the “social history" section from each note and used it as the raw input for further augmentation. Next, based on SDoH data with ICD-10-CM Z Codes^20^, we categorize eviction as a new SDoH, classified under Z59.89, which addresses problems related to housing and economic circumstances. This classification positions eviction alongside other related classes under the Z59 category, as illustrated in Fig. 4. We classify eviction-related categories into 7 specific ones based on two key factors: presence (Absent, Hypothetical, Present, Pending, Mutual Rescission (MR)) and period (Current, History, Future)^27^:

- Eviction Absent: No history or indication of eviction.
- Eviction Present History, Eviction Present Current: Eviction has already been completed. “Current" refers to ongoing eviction impacts within the current natural year, not related to eviction. “History" refers to the past without a specific time or that occurred more than one year ago.
- Eviction Hypothetical: Refers to possible eviction based on hypothetical scenarios.
- Eviction Pending: Eviction has been noticed but is not yet completed.
- Eviction MR History, Eviction MR Current: MR is a legal agreement between the landlord and tenant to terminate a lease. The definitions of “Current" and “History" follow those used for Eviction Present.

In addition to eviction-related categories, we included Tier 1 categories: Homelessness (Z59.0), Inadequate housing (Z59.1), and Lack of adequate food (Z59.4). We also incorporated Tier 2 categories, including Housing instability (Z59.81), Transportation insecurity (Z59.82), Financial insecurity (Z59.86), and Material hardship (Z59.87). In total, we defined 14 classes. For each class, experts provided precise definitions and a few-shot examples to guide data augmentation. Detailed definitions and examples for each category are provided in Supplementary Tables S6 and S7.

Then, as shown in Supplementary Table S10 Algorithm 1, we systematically developed an augmenter for each SDoH class. In our context, an augmenter is essentially an LLM enhanced with an optimized prompt. Once the prompt is fully optimized, the augmenter takes raw notes as input and generates augmented notes that accurately reflect the target SDoH class. For every SDoH class, the process starts by defining the SDoH category and providing raw notes to guide the LLM in generating relevant augmented notes. We employ a targeted HITL procedure to ensure that all augmented notes accurately encode their intended SDoH categories and to iteratively refine augmentation prompts. The objective is to obtain high-fidelity synthetic notes while maintaining a reproducible, model-guided refinement loop. For each SDoH label, we constructed a label-specific augmentation prompt and generated 20 candidate notes using GPT-4o-mini. Domain experts evaluated each note under a strict binary criterion: *correct* (faithfully expresses the target SDoH) or *incorrect/ambiguous*. All incorrect or ambiguous notes were annotated with concise explanations, forming structured feedback for downstream refinement. A prompt was accepted if its human-verified accuracy reached ≥95%. Otherwise, all error cases, together with expert feedback, were provided to a separate LLM to propose an improved prompt. For labels below the threshold, a new batch of 20 notes was generated and re-evaluated. This iterative loop continued for up to three rounds. In practice, 9 labels satisfied the accuracy criterion in the first round, 3 additional labels in the second round, and the remaining 2 labels in the third. Prompts that met the threshold were used to generate the final augmented data, and all notes validated as correct across iterations were directly incorporated into the devset. Across all rounds, experts reviewed 420 unique notes (Round 1: 20 × 14; Round 2: 20 × 5; Round 3: 20 × 2). Three annotators participated, all with clinical and public-health backgrounds drawn from both healthcare systems and academic medical institutions. Each note was independently reviewed by at least two annotators. A note was accepted only under full agreement; any disagreement triggered an “ambiguous” label with a written rationale. Accuracy is calculated as the proportion of correct annotations out of total outputs. If accuracy exceeds 90%, the augmenter is deemed successful for that class. For augmenters that do not meet the accuracy threshold, incorrect cases and feedback are returned to the LLM. The feedback is used to refine the prompt. The updated prompt is re-evaluated, and the cycle continues until the accuracy criterion is satisfied. We conducted three optimization rounds to finalize augmenters for all 14 SDoH classes. For classes with clear distinctions and well-defined criteria (e.g., Tier 1 and Tier 2 SDoH categories), most augmenters reached the desired accuracy within a single iteration. For eviction-related classes, which involved finer granularity and ambiguous definitions, multiple iterations were required. This iterative process significantly improved the accuracy and reliability of the augmenters. Each finalized augmenter demonstrated the capability to consistently generate augmented notes reflecting the target SDoH class. The optimized prompts for all classes are available in the Supplementary Table S8.

### DSPy annotator setting and training process

Inspired by the systematic survey of APO techniques^28^, the practical implementation of APO via DSPy^29^, and our prior work demonstrating structured prompt optimization in healthcare settings^59^, we design our annotator module as a critical component to ensure precise and efficient classification of notes into multiple SDoH categories.

The dataset contains overlapping categories, particularly between Tier 1 and Tier 2, and eviction-related classes. For instance, eviction can lead to homelessness or financial insecurity can lead to eviction, causing ambiguity in multi-class annotation. To address these overlaps, we first used a binary classification to determine whether content is related to eviction, and then divided the dataset into two groups, corresponding to three separate annotators, as outlined in Supplementary Table S10 Algorithm 2. The second annotator handles notes related to eviction-specific categories, including the following 7 labels: Eviction Absent, Eviction Present History, Eviction Present Current, Eviction Hypothetical, Eviction Pending, Eviction MR History, Eviction MR Current. The third annotator manages notes that do not directly pertain to eviction, covering the remaining 7 labels: Homelessness, Inadequate Housing, Lack of Adequate Food, Housing Instability, Transportation Insecurity, Financial Insecurity, and Material Hardship. This division ensures better class separation and significantly improves annotation accuracy.

For each annotator of the three, we implemented the training process using DSPy, which provides modular abstractions for programmatic prompt optimization. Below, we describe the model signature, prompting modules, optimization loop, and evaluation procedure in sufficient detail for reproduction.

We defined an AnnotatorSignature specifying the input–output behavior of the annotator: the input is a patient social history note (fact), and the output is a structured SDoH label (answer). The prompt template onsists of: (i) a high-level instruction describing the annotation task, (ii) the full label set with definitions. Take the Step 2 (eviction subcategories) as an example, the exact label descriptions follow the rules stated in Supplementary Table S6. (e.g., distinctions between *present*, *pending*, *hypothetical*, and temporal modifiers such as *current* vs. *history*). This template is statically included in the answer field of the signature and is optimized automatically through DSPy.

We instantiate a DSPy module, Annotator, which wraps a CoT-enabled predictor: dspy.ChainOfThought(AnnotatorSignature). This module generates both an intermediate rationale and a final label. All experiments used the same seed to ensure deterministic sampling during optimization.

We used BootstrapFewShotWithRandomSearch as the optimizer for Automatic Prompt Optimization (APO). At each round, the optimizer: (1) samples candidate demonstrations from the training data, (2) constructs alternative prompt variants, (3) evaluates these variants using the evaluation metric, and (4) selects the best-performing prompt configuration to seed the next round. We set the number of optimization rounds to 5. Unlike standard few-shot prompting, the Bootstrapped Random Search explicitly explores prompt variations and jointly tunes the demonstrations and prompt fields. This makes the optimization effective even under cold-start conditions (e.g., eviction classification in Step 2).

Following DSPy best practices, all prompt variants were evaluated with the AnswerExactMatch metric, which enforces exact string equality between the predicted label and the gold annotation. This ensures a strict objective function that favors consistent, category-aligned outputs. The optimizer maximizes this metric to produce an APO-enhanced annotator program.

Training data are converted into DSPy Examples (input–output pairs). The optimizer then compiles the Annotator module into an optimized program:

1 $${\mathtt{compiled}}\_{\mathtt{annotator}}={\mathtt{teleprompter.compile(Annotator(),\; trainset)}}.$$

The resulting compiled system contains the optimized prompt, refined few-shot examples, and CoT reasoning logic. This compiled model is saved and used during inference.

We evaluate the optimized program on a devset using the DSPy Evaluate module, with multi-thread execution enabled. The evaluator returns instance-level correctness and label-specific accuracies.

The same pipeline can be readily adapted to other SDoH categories by modifying the label definitions and task description in the initial prompt and by supplying the corresponding train and dev sets for optimization.

For each DSPy program, the trainset consists of data generated by the augmenter, specifically GPT-rewritten notes that have been verified by human evaluators. The trainset is carefully balanced, with 8 examples for each label category to ensure equal representation across all classification targets.

For the devset, we designed a more comprehensive evaluation to ensure the model’s robustness and performance, with each label category represented by 48 examples. The devset includes three groups of data:

- Synth clinical notes: Similar to the trainset, this group consists of notes generated by GPT-4o-mini and verified by human experts, used to test the model’s ability to generalize to data created by the augmenter.
- MIMIC-IV^19^ clinical notes: To assess the model’s performance on real-world data, we included raw notes from the MIMIC dataset. These notes primarily cover Tier 1 and Tier 2 SDoH categories but do not initially contain any references to eviction. To introduce eviction-related content, we first used a set of keywords (listed in Supplementary Table S11) to filter the raw notes. For cases where this filtering process did not yield sufficient results, we employed human experts to rewrite a subset of these raw notes. The rewriting process was done carefully, ensuring that the original structure and characteristics of the notes were preserved while selectively adding eviction information. This rewriting step is not standard augmentation but is introduced to bridge the stylistic gap between synthetic notes and real-world MIMIC documentation, enabling realistic evaluation while preserving annotation accuracy through expert validation.
- PMC-Patient^31^ case reports: The third group comprises raw notes from PMC articles, following a similar strategy as the MIMIC raw notes. These notes were rewritten by humans to include targeted eviction-related scenarios without altering the inherent structure and characteristics of the original text.

By testing the model across these three distinct groups, we ensure that it can effectively handle both augmented and real-world data, as well as notes that have been selectively modified to introduce eviction scenarios. This diverse evaluation helps validate the model’s performance and generalizability across various SDoH contexts.

### Leveraging augmenters and annotators for fine-tuning open-source models

Our fine-tuning process leverages the augmenter-annotator pipeline, which is instrumental in generating large volumes of high-quality labeled data. Label-specific augmenters were used to generate augmented notes tailored to the target labels. Each augmenter outputs synthetic data related to a specific SDoH category. The output from the augmenters was further annotated by the multi-label annotator. This annotator assigns the correct label along with corresponding reasoning.

In our fine-tuning process, we iteratively improved the model’s performance through two phases. Initially, we exclusively used GPT-augmented sentences, which performed well on shorter texts but showed limited generalization on PMC long notes, highlighting the need for more diverse data. In the second phase, we enriched the training set by adding 30% PMC long notes, significantly enhancing the model’s ability to handle complex inputs.

For the selection of open-source models, we conducted extensive experiments with multiple model architectures. We fine-tuned various models including BERT-based models (bert_base_cased-FT, biobert-v1.1-FT), domain-specific models (Bio_ClinicalBERT-FT), and their SBDH-adapted variants (sdh_bert_base_cased-FT, sdh_biobert-v1.1-FT, etc.). We also explored LLMs such as LLaMA-3.1-8B-FT, LLaMA-3.2-3B-FT, Qwen2.5-7B-FT, and Qwen2.5-3B-FT, along with their SBDH-adapted versions. While all models demonstrated improvements after fine-tuning, the larger models (particularly the 7B and 8B variants) showed superior capacity for generalization and performance compared to the 3B-level models, which had more limited gains. This comprehensive approach allowed us to identify the most effective model architectures for our specific task.

### Experimental settings

First, for the main experimental setup, we conducted a comprehensive evaluation across three sequential steps (Step 1: Binary Classification, Step 2: Eviction Multi-Class Classification, and Step 3: Non-Eviction Multi-Class Classification). For each model, we performed 5 independent runs to calculate 95% confidence intervals, ensuring the statistical reliability of our results. These five runs per model also served as the sample basis for statistical inference: for each model-dataset pair, we obtained a vector of five performance scores. For the p-values reported in Table 1, we applied two-sided independent two-sample t-tests comparing these scores against those of a single designated baseline model (GPT-4o-APO). The 95% confidence intervals were likewise computed from these five scores using Student’s t-distribution with degrees of freedom equal to the number of runs minus one.The exact p-values corresponding to all statistical comparisons are provided in Supplementary Information (Exact p-values) for completeness. For language models like GPT-4o and GPT-4o-mini, we initially used temperature = 0 for the annotation task to minimize uncertainty. However, to compute reliable confidence intervals, we performed 4 additional runs with temperature = 0.5. For models utilizing DSPy frameworks and other LM/LLM architectures, we maintained consistency by using different random seeds across the 5 runs. Throughout all experiments, we kept the fine-tuning process consistent, with identical fine-tuning parameters across all models and training set sizes. To ensure compliance with MIMIC-IV data usage requirements, we exclusively used the Azure OpenAI service for GPT-4o and GPT-4o-mini, with human review explicitly disabled, following PhysioNet’s guidelines for responsible use of credentialed data. We introduce the detailed experimental settings in the Supplementary Information (Fine-tuning Settings). Each model was evaluated on three distinct devsets (Synth, Mimic, and PMC), with the average performance calculated by simply taking the arithmetic mean across these three datasets. This approach allows us to assess model generalization across different data distributions. The overall performance metric was derived from the best-performing model configuration for each step. Importantly, we consider a prediction correct only when Step 1 (Binary Classification) is correct, and either Step 2 (Eviction Multi-Class Classification) or Step 3 (Non-Eviction Multi-Class Classification) is also correct for the same clinical note. This evaluation approach reflects the cascading nature of our classification task, where Step 1 determines whether a note contains eviction class, while Steps 2 and 3 classify non-overlapping subsets of notes. This evaluation framework ensures that models must perform well across the entire classification pipeline to achieve high overall scores, providing a realistic assessment of their practical utility in clinical document analysis.

Second, for the training data size impact experiment, we tested a diverse set of models, including traditional LMs (BERT variants) and LLMs (Llama and Qwen families), on both Eviction and Non-Eviction Multi-Class Classification. We used five different training set sizes (200, 1000, 3000, 5000, and 10000 samples) to establish clear performance trends. As the dataset size increased, the number of examples in each class increased proportionally. The datasets for BERT-based models contained only labels without reasoning, while LLM datasets included both labels and reasoning. Each training set was run only once, with consistent fine-tuning parameters across all experiments.

Third, for the reasoning in trainset experiment, we trained LLMs (Llama-3.1-8B, Llama-3.2-3B, Qwen2.5-7B, and Qwen2.5-3B) using two distinct training approaches: (1) With Reasoning: Models were trained on datasets containing both classification labels and explicit reasoning steps. (2) Without Reasoning: Models were trained on identical datasets but with only classification labels (reasoning steps removed). All models were evaluated on both Eviction and Non-Eviction Multi-Class tasks using Micro-F1 scores as the primary performance metric. All fine-tuning hyperparameters were kept consistent between the with-reasoning and without-reasoning training conditions to isolate the specific impact of reasoning annotations.

### Metrics

Following Yao et al. (2023)^27^, we calculate the confusion matrix C for both tasks, which are True Positives (TP), True Negatives (TN), False Positives (FP), and False Negatives (FN) for each class. We use both Micro-F1 and Macro-F1 in our evaluation. We also report the Matthews Correlation Coefficient (MCC) score, a metric that yields a high score only if the prediction yields good results in all four confusion matrix categories (TP, TN, FP, and FN).

### Declaration of generative AI and AI-assisted technologies in the writing process

During the preparation of this work, the author(s) used ChatGPT to improve the language and readability. Following the use of this tool, the author(s) carefully reviewed and edited the content as necessary and take full responsibility for the final version of the manuscript.

## Supplementary information

Supplementary Information

## Data Availability

Synth: https://huggingface.co/datasets/youxiazhao/eviction-devset-synth PMC-Patients: https://huggingface.co/datasets/youxiazhao/eviction-devset-pmc Only individuals who have signed the MIMIC Data Use Agreement may contact us to obtain the MIMIC-based version of the dataset.

## Code availability

The code is publicly available on Github: https://github.com/youxiazhao/sdoh_annotation.
