# Supplementary material for: SynthEHR-eviction: enhancing eviction SDoH detection with LLM-augmented synthetic EHR data
Source: NPJ Digit Med. 2026 Feb 27;9:292. doi: 10.1038/s41746-026-02473-0 (PMC13066574; doi:10.1038/s41746-026-02473-0)
Supplement: Supplementary file 1 — Supplementary Information [file 41746_2026_2473_MOESM1_ESM.pdf]

Supplementary Information for SynthEHR-Eviction: Enhancing Eviction SDoH Detection with LLM-Augmented Synthetic EHR Data

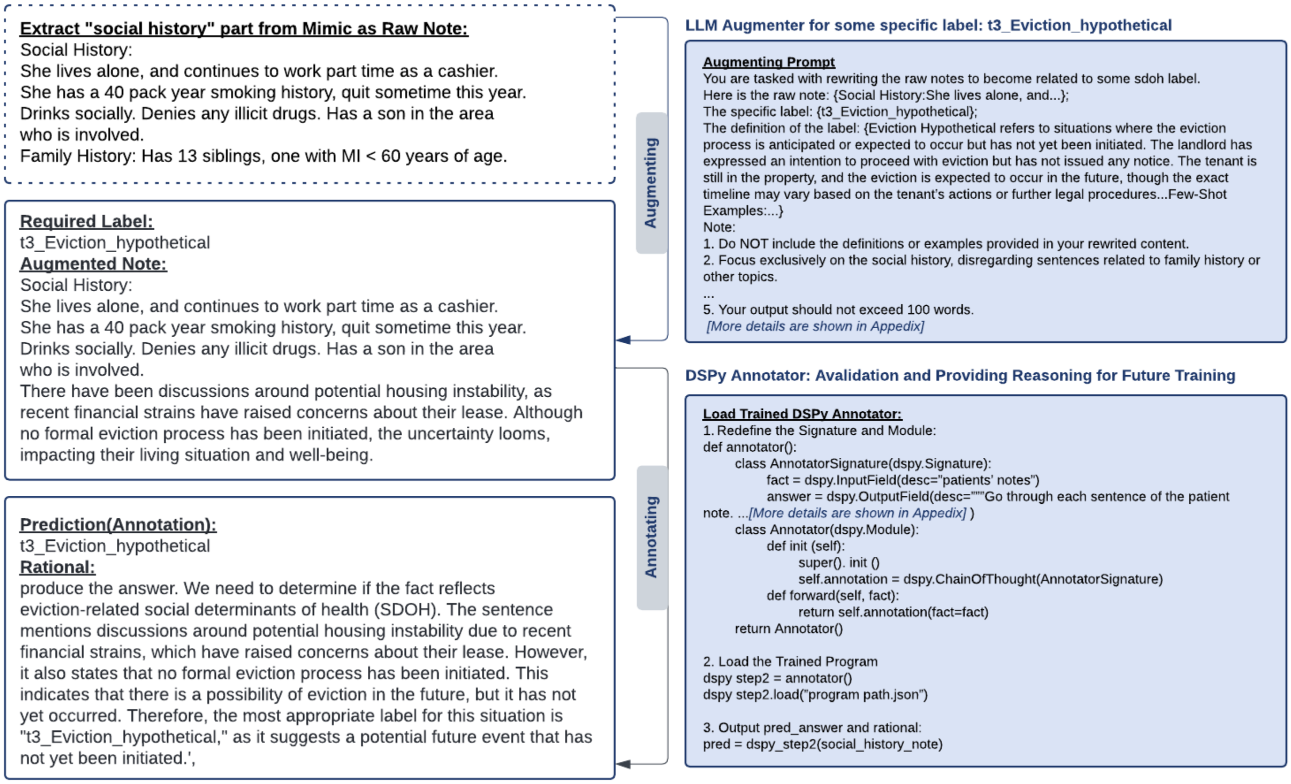

Figure S1. An example of label-specific note augmentation and subsequent annotation for the eviction-related SDoH label. The social history section is first extracted from raw MIMIC clinical notes and used as input to a label-specific LLM augmenter (here, `t3_Eviction_hypothetical`), which rewrites the note to reflect eviction-related social context while preserving original clinical details. The augmented note is then validated and annotated by a DSPy-based annotator, producing a structured eviction-related label and rationale for downstream training.

	Step 2: Eviction Multi-Class			Step 3: Non-Eviction Multi-Class		
Training Set	GPT-4o-APO	Llama-3.1-8B-FT	Qwen2.5-7B-FT	GPT-4o-APO	Llama-3.1-8B-FT	Qwen2.5-7B-FT
100% Synth	0.835(0.692)	0.797(0.606)	0.800(0.558)	0.863(0.789)	0.870(0.800)	0.864(0.793)
70% Synth 30% PMC	0.915(0.929)	0.913(0.888)	0.912(0.860)	0.911(0.922)	0.929(0.942)	0.925(0.944)

Table S1. Comparison of model performance across different training data compositions.

Training Set Composition Our initial training approach utilized purely synthetic data for model training. However, this configuration demonstrated suboptimal performance on PMC note evaluations, revealing limitations in the synthetic data's ability to capture the nuances of real-world medical documentation. To address this limitation, we modified our training set composition by replacing 30% of synthetic data with actual PMC notes. This hybrid approach yielded substantial improvements across different experimental settings. As shown in Supplementary Table S1, the introduction of PMC data led to an increase of 0.237 in Micro-F1 score on the PMC devset in our DSPy-based experiments, while achieving an overall average improvement of 0.08 across all evaluation metrics. Similarly, in the LLM fine-tuning experiments, such as with Qwen2.5-7B-FT, the hybrid training set demonstrated even more pronounced benefits, with performance on PMC-specific tasks improving by 0.302 while overall performance increased by 0.112.

To further examine whether the observed gains depend on a specific Synth–PMC mixing ratio, we conducted an additional sensitivity analysis by varying the proportion of PMC data in the training set. We focus on the Step 2 eviction multi-class

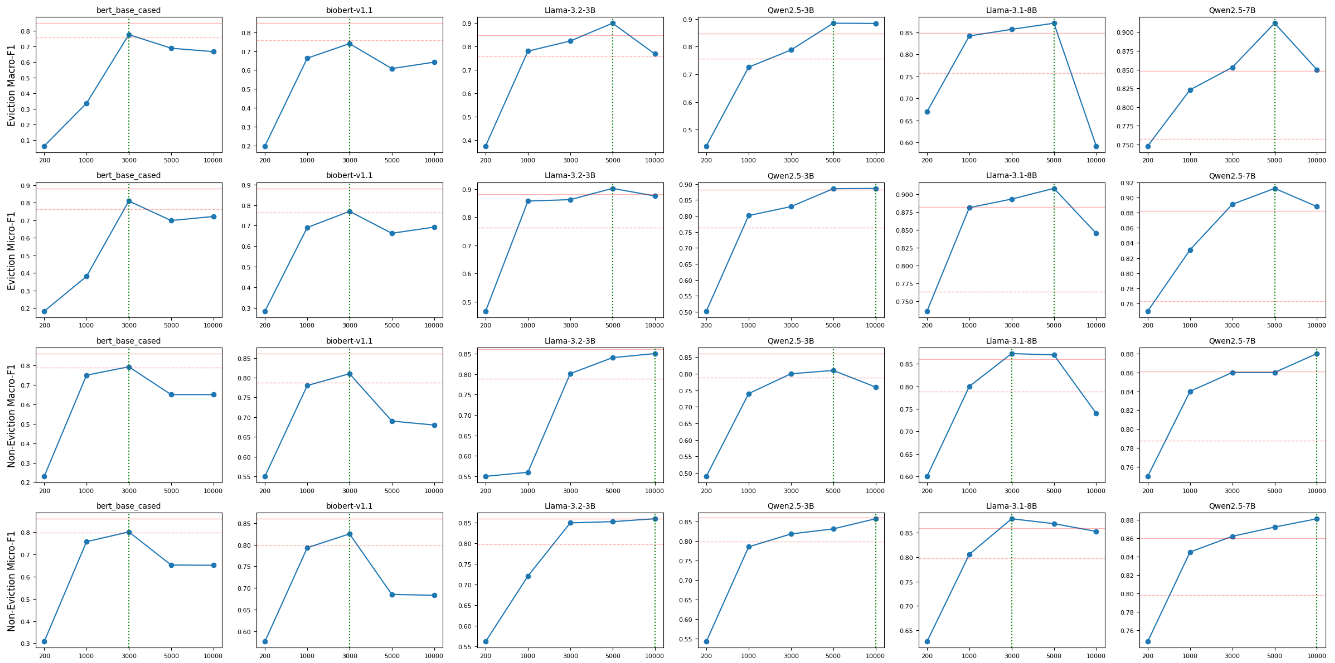

Figure S2. Impact of training set size on model performance across eviction and non-eviction classification tasks. Each subplot illustrates how model performance varies with different training set sizes (x-axis) across four classification scenarios: eviction (top two rows) and non-eviction (bottom two rows), evaluated using two metrics—Macro-F1 (first and third rows) and Micro-F1 (second and fourth rows). The y-axis represents the corresponding F1 score. Green vertical dashed lines indicate the training data size at which each model achieves its highest performance. Red solid and Red dashed horizontal lines denote the performance of GPT-4o-APO and GPT-4o-mini-APO, serving as a reference here.

classification task and fine-tune a representative model (Qwen2.5-7B) to isolate the effect of training data composition. Across all settings, the total training set size is fixed at 5,000 samples, while the proportion of PMC data is varied among {0%, 10%, 20%, 30%, 40%}, with the remainder drawn from synthetic data. Importantly, only the *trainset composition* is modified in this experiment. All evaluations are conducted on the same held-out devsets (Synth, MIMIC, and PMC) used throughout the paper, ensuring direct comparability across different mixing ratios.

PMC Ratio	Δ Macro-F1	Δ Micro-F1
0%	-2.80%	-1.18%
10%	**+0.58%**	**+0.07%**
20%	+0.02%	-0.55%
30%	–	–
40%	-1.71%	-2.43%

Table S2. Relative performance changes compared to the 30% PMC setting for Step 2 eviction multi-class classification using Qwen2.5-7B, with total training size fixed at 5,000 samples.

Overall, performance remains relatively stable across PMC ratios between 10% and 30%, with only modest fluctuations. While the 10% PMC setting yields a slightly higher Macro-F1, the difference is small and no monotonic trend is observed as the proportion of real data increases. In contrast, both purely synthetic training (0%) and higher PMC dominance (40%) lead to noticeable performance degradation, suggesting diminishing returns when real data constitutes either too small or too large a fraction of the training set.

Based on these observations, we retain the 30% PMC setting in all main experiments to ensure consistency. Importantly, this analysis indicates that our conclusions do not rely on a narrowly tuned mixing ratio, supporting the robustness of the proposed synthetic–real training strategy.

Exact p-values To complement the statistical results summarized in the main tables, we report the exact p-values for all model comparisons in Supplementary Table S3.

Model	Synth p-value	MIMIC p-value	PMC p-value	Avg p-value
**Step 1: Binary Classification**				
GPT-4o-mini	6.97×10^{-8}	4.96×10^{-11}	5.52×10^{-6}	5.92×10^{-10}
GPT-4o-mini-APO	1.11×10^{-6}	2.34×10^{-6}	6.59×10^{-6}	2.04×10^{-7}
GPT-4o	5.91×10^{-4}	1.33×10^{-6}	7.86×10^{-8}	2.02×10^{-7}
SynthEHR-Eviction (bert_base_cased)	8.37×10^{-3}	5.52×10^{-5}	5.51×10^{-6}	2.30×10^{-5}
SynthEHR-Eviction (biobert-v1.1)	1.17×10^{-3}	6.47×10^{-2}	4.62×10^{-3}	1.96×10^{-1}
SynthEHR-Eviction (Bio_ClinicalBERT)	8.51×10^{-3}	9.65×10^{-6}	2.13×10^{-3}	6.53×10^{-5}
**Step 2: Eviction Multi-Class**				
GPT-4o-mini	5.02×10^{-11}	2.09×10^{-10}	1.08×10^{-7}	1.42×10^{-10}
GPT-4o-mini-APO	7.60×10^{-3}	1.68×10^{-5}	6.14×10^{-4}	1.65×10^{-4}
GPT-4o	2.06×10^{-7}	4.88×10^{-9}	8.86×10^{-7}	1.69×10^{-8}
SynthEHR-Eviction (bert_base_cased)	8.40×10^{-6}	0.845	1.26×10^{-4}	1.18×10^{-4}
SynthEHR-Eviction (biobert-v1.1)	1.17×10^{-3}	8.10×10^{-5}	5.99×10^{-5}	3.31×10^{-5}
SynthEHR-Eviction (Bio_ClinicalBERT)	1.54×10^{-5}	4.31×10^{-4}	1.41×10^{-7}	2.54×10^{-7}
SynthEHR-Eviction (LLama-3.1-8B-FT)	-	6.81×10^{-3}	-	-
SynthEHR-Eviction (LLama-3.2-3B-FT)	-	-	-	-
SynthEHR-Eviction (Qwen2.5-7B-FT)	-	6.08×10^{-4}	-	-
SynthEHR-Eviction (Qwen2.5-3B-FT)	-	-	-	-
**Step 3: Non-Eviction Multi-Class**				
GPT-4o-mini	2.95×10^{-4}	6.15×10^{-8}	3.49×10^{-6}	9.10×10^{-8}
GPT-4o-mini-APO	-	2.88×10^{-3}	-	1.60×10^{-2}
GPT-4o	2.96×10^{-3}	2.95×10^{-2}	2.19×10^{-3}	2.42×10^{-4}
SynthEHR-Eviction (bert_base_cased)	1.87×10^{-3}	1.67×10^{-4}	2.07×10^{-3}	1.39×10^{-3}
SynthEHR-Eviction (biobert-v1.1)	3.05×10^{-3}	1.28×10^{-3}	1.87×10^{-3}	3.05×10^{-3}
SynthEHR-Eviction (Bio_ClinicalBERT)	2.74×10^{-3}	1.85×10^{-8}	1.44×10^{-8}	8.44×10^{-9}
SynthEHR-Eviction (LLama-3.1-8B-FT)	1.28×10^{-3}	8.02×10^{-2}	2.30×10^{-2}	2.43×10^{-2}
SynthEHR-Eviction (LLama-3.2-3B-FT)	7.71×10^{-2}	2.42×10^{-3}	-	-
SynthEHR-Eviction (Qwen2.5-7B-FT)	1.87×10^{-3}	-	-	2.97×10^{-2}
SynthEHR-Eviction (Qwen2.5-3B-FT)	9.28×10^{-3}	6.22×10^{-4}	4.83×10^{-2}	2.35×10^{-2}

Table S3. Exact p-values (all comparisons relative to GPT-4o-APO baseline).

Fine-tuning Settings Our fine-tuning methodology was tailored to each model architecture to optimize performance while maintaining computational efficiency. For large language models (LLaMA and Qwen variants), we employed Unsloth[?], a parameter-efficient fine-tuning framework that enables significantly faster training and inference compared to conventional methods.

For LLaMA and Qwen models, we implemented the following configuration using Unsloth:

- Maximum sequence length of 1024 tokens with 4-bit quantization (load_in_4bit=True) to reduce memory usage
- LoRA (Low-Rank Adaptation) with rank (r) of 16 and alpha value of 16 Target modules included query, key, value projections ('q_proj', 'k_proj', 'v_proj', 'o_proj', 'gate_proj', 'up_proj', 'down_proj')
- LoRA dropout set to 0 and bias configuration set to 'none' for optimized performance
- Learning rate of 2e-4 with a linear scheduler
- Training for 2 epochs with gradient accumulation steps of 4
- Warmup steps of 5 and weight decay of 0.01
- Per-device batch size of 2 with AdamW 8-bit optimizer

Our training dataset was split into training and test sets with an 80/20 ratio (test_size=0.2), using a seed of 42 for reproducibility. For data formatting, we employed a chat template structure with a system prompt instructing the model to act as a healthcare annotator specifically focused on eviction-related content. This approach helped align the model's responses with our annotation requirements.

For BERT-based models (bert_base_cased, biobert, etc.), we utilized the standard fine-tuning approach from Hugging Face's Transformers library. These models were trained with the same learning rate (2e-4) and epochs (2) as the LLMs for consistency, but with appropriate modifications to accommodate their architectural differences. The fine-tuning was performed on a Tesla T4 GPU with 14.748 GB of memory.

The hyperparameters for each model were determined through preliminary experiments, with optimal configurations selected based on Micro-F1 scores for our target SBDH categories. This comprehensive approach allowed us to identify the most effective model architectures while maintaining computational efficiency through parameter-efficient fine-tuning techniques.

All models, including the 3B and 7B/8B variants, were trained using an epoch-based schedule with identical optimization hyperparameters, the same number of epochs (2), and the same training set for each task (5,000 examples for Step 2 eviction multi-class classification and 3,000 examples for Step 3 non-eviction multi-class classification). A training step corresponds to a single optimizer update; therefore, the total number of optimizer update steps is identical across model sizes. Per-device batch size and gradient accumulation steps were also kept constant, resulting in comparable effective batch sizes across scales.

The only difference between the "with reasoning" and "without reasoning" conditions lies in the inclusion of a textual rationale appended to the output. Using the same tokenizer and a fixed maximum sequence length of 1024 tokens, adding rationales increases the average sequence length and the fraction of truncated sequences. Importantly, truncation consistently affects only the tail of the appended rationale and is applied identically across all model sizes. As a result, differences in training budget, optimization steps, or truncation behavior cannot account for the observed performance differences across model sizes.

Overall performance for extension of Table 1 The overall performance metric was derived from the best-performing model configuration for each step. Importantly, we consider a prediction correct only when Step 1 (Binary Classification) is correct, and either Step 2 (Eviction Multi-Class Classification) or Step 3 (Non-Eviction Multi-Class Classification) is also correct for the same clinical note. This evaluation approach reflects the cascading nature of our classification task, where Step 1 determines whether a note contains eviction class, while Steps 2 and 3 classify non-overlapping subsets of notes. This evaluation framework ensures that models must perform well across the entire classification pipeline to achieve high overall scores, providing a realistic assessment of their practical utility in clinical document analysis. Results are shown in Supplementary Table S14.

Performance Variations Across Different Devsets Our comprehensive analysis compared performance across three distinct datasets: Synth, MIMIC, and PMC in the Eviction Multi-Class task (Figure 1). The Synth dataset consistently demonstrated the most stable performance characteristics, exhibiting high and uniform $F1$ scores across eviction classes. Similarly, the MIMIC dataset showed minimal variability, with stable and reliable performance across most classes. In contrast, the PMC dataset demonstrated the most significant performance heterogeneity, characterized by the widest performance ranges and lower median scores. This performance degradation aligns with the trends observed in Table 1, where models exhibited substantially lower micro-F1 scores on PMC data compared to Synth and MIMIC datasets. These results highlight notable inter-dataset variations, particularly emphasizing the challenges posed by PMC notes.

Interpretation of Inter-Dataset Variability The performance discrepancies between datasets, particularly the pronounced heterogeneity in PMC results, can be attributed to both structural differences in the clinical notes and model limitations. Specifically, PMC notes tend to be significantly longer and more detailed, leading to truncation due to our 512-token input limit, which likely eliminates crucial contextual information. Moreover, unlike the Synth and MIMIC datasets, where eviction-related information is often explicitly stated and follows a structured format, PMC notes embed relevant details within complex, narrative clinical discussions. This structural complexity complicates the model's ability to generalize, especially when it has been primarily trained on more structured datasets.

Evaluation Under Realistic Prevalence Conditions To assess the feasibility of our pipeline under realistic prevalence conditions, we conducted an additional experiment using a highly imbalanced evaluation set derived from MIMIC. We randomly sampled 2,000 clinical notes and extracted their social history sections. Notes with extremely short or non-informative social history were removed, resulting in a final set of 1,829 notes. Each note was manually reviewed, and only 28 notes (approximately 1.5%) were verified as eviction-related, reflecting the extreme sparsity of eviction signals in real-world clinical documentation.

We evaluated the same two-step pipeline used in the main experiments. Step 1 performs binary classification to determine whether a note contains eviction-related content. Step 2 performs fine-grained eviction classification and is applied only to notes identified as eviction-positive in Step 1. This setup allows us to separately assess false positive control and fine-grained classification performance under realistic prevalence.

Supplementary Table S4 reports performance for binary eviction detection on the full set of 1,829 notes. DSPy-based annotators and fine-tuned open-source models achieve strong performance despite extreme class imbalance, indicating effective control of false positives. Supplementary Table S5 reports performance on the 28 eviction-positive notes identified in the dataset. Although the sample size is small, fine-tuned open-source models and DSPy annotators substantially outperform base GPT models, demonstrating improved discrimination among eviction subtypes once relevant notes are identified.

Model	n_samples	Micro-F1	Macro-F1
GPT-4o	1829	0.990	0.865
GPT-4o-mini	1829	0.957	0.698
DSPy-4o	1829	**0.996**	**0.936**
DSPy-4o-mini	1829	0.970	0.742
Llama-3.1-8B-FT	1829	0.846	0.531
Qwen2.5-7B-FT	1829	0.992	0.858
Llama-3.2-3B-FT	1829	0.730	0.463
Qwen2.5-3B-FT	1829	0.959	0.586

Table S4. Step 1 binary eviction detection under realistic prevalence (1.5% positives).

Model	n_samples	Micro-F1	Macro-F1
GPT-4o	28	0.393	0.393
GPT-4o-mini	28	0.429	0.344
DSPy-4o	28	0.750	0.646
DSPy-4o-mini	28	0.464	0.420
Llama-3.1-8B-FT	28	0.679	0.418
Qwen2.5-7B-FT	28	0.714	0.709
Llama-3.2-3B-FT	28	**0.786**	0.644
Qwen2.5-3B-FT	28	0.750	**0.716**

Table S5. Step 2 fine-grained eviction classification on eviction-positive notes under realistic prevalence.

Illustrative Extension to Other SDoH Domains To clarify which components of the proposed pipeline support reuse beyond eviction-specific settings, we outline an illustrative extension to another SDoH domain (e.g., food insecurity) following the same modular workflow shown in Figure 3.

Step 1: Expert-guided schema definition. Domain experts define a small set of clinically meaningful categories relevant to the target SDoH domain (e.g., food insecurity). This schema specification represents the primary source of domain knowledge and is typically lightweight compared to full-scale manual annotation.

Step 2: Label-specific augmentation with HITL. For each category, a label-specific LLM augmenter is initialized to generate synthetic clinical notes under the expert-defined schema. A small subset of generated examples is reviewed by experts, who provide targeted feedback to iteratively refine the augmenters through a human-in-the-loop (HITL) process. This step ensures clinical plausibility while avoiding large annotation overhead.

Step 3: Constrained and diversity-aware augmentation. The refined augmenters generate synthetic notes under structural constraints while incorporating linguistic variability characteristic of real-world EHR documentation. This enables coverage of rare, temporally nuanced, or implicitly expressed scenarios without requiring domain-specific rewriting procedures.

Step 4: DSPy-based annotation with limited expert data. A small expert-validated train/dev set is used to configure DSPy-based annotators by specifying task signatures, multi-step decision structures, and evaluation objectives. This step does not require large-scale labeled data and is reusable across domains with minimal modification.

Step 5: Large-scale annotation and fine-tuning. The optimized annotators are then applied to the augmented corpus to produce a large-scale labeled dataset, which can be used to fine-tune open-source language models for downstream deployment.

Importantly, the pipeline is not eviction-specific. Domain knowledge enters primarily through (i) the expert-defined label schema and (ii) limited HITL feedback during augmenter refinement. The remaining components—including augmentation

orchestration, constrained synthetic generation, DSPy-based prompt optimization and annotation, and the downstream fine-tuning workflow—are task-agnostic and reusable across SDoH domains.

Examples of Patient Notes Sample patient notes from different training datasets are provided in Supplementary Table S9 to illustrate the diversity of source notes used in training and test.

To clarify the contribution of the rewriting stage, we provide a representative example comparing an original excerpt with its eviction-focused rewritten version.

Original note:

> *Social History: Lives with daughter, on disability, no current tobacco use (quit 15 years ago, smoked 30 years prior), very occasional EtOH.*

Rewritten note (eviction-related):

> *Social History: Lives with daughter now, evicted from the rented house this year, on disability, no current tobacco use (quit 15 years ago, smoked 30 years prior), very occasional EtOH.*

This example illustrates the design principle of the expert rewriting stage: the label-specific augmenter preserves the original note structure, clinical details, and writing style, while minimally introducing eviction-related information consistent with the target SDoH label. No unrelated social or medical attributes are altered or removed. This controlled augmentation strategy aims to inject task-relevant signals without distorting the original clinical context.

Expert Validation of Test-Set Fidelity and Realism We conducted expert review on all 616 test instances to directly validate note fidelity and clinical realism. The review was performed by one expert MPH annotator, a native English speaker with 8+ years of EHR annotation experience, including 4+ years on SDoH and 4+ years specifically on eviction-related SDoH. The expert followed a simple guideline: (i) verify that the note is clinically plausible and consistent with EHR documentation style, (ii) verify that eviction-related statements are coherent and non-contradictory, and (iii) verify that the assigned label is supported by explicit evidence in the text (with temporality and mutual rescission handled according to our definitions). All 616 test notes passed this expert validation (100%).

External validation on a real-world institutional dataset (REC). We added an external evaluation using the VA–Eviction dataset from prior work[?]. We denote this dataset as *Real-Eviction-Clinical (REC)*. REC contains fully real clinical notes from a new institution, so it directly tests how models behave on messy EHR text beyond our synthetic and semi-synthetic settings. We report results in the revised manuscript, and we summarize the dataset, label mapping, and evaluation protocol here for transparency.

1. REC annotations and our 7-label taxonomy. The VA–Eviction dataset provides two complementary annotations for each note: (i) *Eviction period* with values *future*, *irrelevant*, *current*, *uncertain*, *history*, and *no present*; and (ii) *Eviction presence* with values *absent*, *uncertain*, *pending*, *mutual rescission*, and *present*. Our framework uses a single-label taxonomy with seven eviction-related classes: `Eviction_absent`, `Eviction_hypothetical`, `Eviction_mr_current`, `Eviction_mr_history`, `Eviction_pending`, `Eviction_present_current`, and `Eviction_present_history`. Since REC uses a two-axis scheme while our task uses a single label, we harmonize labels with a deterministic mapping.

2. Deterministic mapping from VA (period, presence) to our 7 labels. We map *period* = *irrelevant* to `Eviction_absent`, since the eviction mention is not about the patient (for example, "patient has to evict his son"). For *presence* = *absent*, we map to `Eviction_absent`. For *presence* = *pending*, we map to `Eviction_pending`, since this indicates initiated but unresolved proceedings in the VA scheme. For *presence* = *mutual rescission*, we map by time: *period* = *current* maps to `Eviction_mr_current`, and *period* $\in$ *history, no present* maps to `Eviction_mr_history`; if *period* = *uncertain*, we map to `Eviction_mr_history` as a conservative default. For *presence* = *present*, we map by time: *period* = *current* maps to `Eviction_present_current`, and *period* $\in$ *history, no present* maps to `Eviction_present_history`; if *period* = *uncertain*, we map to `Eviction_present_history` as a conservative default. For remaining uncertain-risk cases, we map to `Eviction_hypothetical`: if *presence* = *uncertain* (and no rule above applies) or if *period* = *future*, we map to `Eviction_hypothetical`, since it best matches "anticipated or uncertain eviction risk" in our taxonomy. This mapping keeps the key clinical distinctions in our definitions (present vs. pending vs. hypothetical, current vs. history, and mutual rescission), and it makes VA-specific categories explicit and reproducible.

3. External evaluation protocol (REC-only vs. REC + SynthEHR-Eviction). We train and evaluate models only on REC notes under two regimes. In *REC-only fine-tuning*, we fine-tune on the REC train split. In *REC + SynthEHR-Eviction*, we fine-tune on the REC train split plus our synthetic training data. We keep the same model family and the same fine-tuning setup within each comparison so the effect is isolated to adding SynthEHR-Eviction. We report Macro-F1 on the REC evaluation split, with mean and standard deviation across repeated runs.

SDoH Class	Definition
Eviction_present_current	1. Eviction Present refers to the state where the eviction process has already been fully concluded, and the tenant has been legally removed from the property. All legal proceedings, such as notices and hearings, have been completed, and the tenant no longer has access to the property.<br>- Consideration: When generating the note, it should mention that the eviction has already taken place, and the tenant is no longer living at or has access to the property.<br>2. Current refers to events related to eviction that happened currently or recently, with specificity about the timing. This includes cases where the eviction event occurred in the current year (e.g., ”this year,” ”last month”, ”last week”, ”several months ago,” or ”a few months ago”, ”recently”...).<br>- Consideration: The note should mention that the event was in the current year, providing some specific time reference.
Eviction_present_history	1. Eviction Present refers to the state where the eviction process has already been fully concluded, and the tenant has been legally removed from the property. All legal proceedings, such as notices and hearings, have been completed, and the tenant no longer has access to the property.<br>- Consideration: When generating the note, it should mention that the eviction has already taken place, and the tenant is no longer living at or has access to the property.<br>2. History refers to events related to eviction that happened in the past, but with less specificity about the timing. This includes cases where the eviction event occurred in the distant past (e.g., ”last year,” ”several years ago,” or ”a few years ago”), or where the mutual rescission agreement or eviction action itself isn’t explicitly stated.<br>- Consideration: The note should mention that the event was in the past, providing some general time reference, but without specifying the exact date or events.
Eviction_pending	Eviction Pending refers to a situation where eviction proceedings have been initiated but are not yet complete. The tenant is still in the property, and there is still an opportunity for negotiation, remediation, or resolution before a final court decision or physical removal occurs. This state indicates that while the eviction process has started, the outcome is still undecided, and there is a potential for the tenant to address the issue and avoid eviction.<br>- Distinction from Completed Eviction: Unlike a completed eviction where the tenant has already been removed, eviction-pending indicates that the tenant has received a notice, but there has been no final court order or physical removal. The tenant may negotiate, pay overdue rent, or comply with other conditions to potentially stop the eviction process.<br>Few-Shot Examples:<br>- “The tenant received an eviction notice recently, but negotiations with the landlord to pay overdue rent are still ongoing.”<br>- “A few months ago, the landlord filed for eviction due to nonpayment, but the case is still pending in court, giving the tenant a chance to settle.”<br>- “Currently, the tenant is under an eviction notice but is working with a housing advocate to resolve the issue before the court date.”
Eviction_hypothetical	Eviction Hypothetical refers to situations where the eviction process is anticipated or expected to occur but has not yet been initiated. The landlord has expressed an intention to proceed with eviction but has not issued any notice. The tenant is still in the property, and the eviction is expected to occur in the future, though the exact timeline may vary based on the tenant’s actions or further legal procedures.<br>- Time Frame: ”hypothetical” in this context refers to actions or plans related to eviction that are expected to occur soon. This could include statements like ”in the coming weeks,” ”next month,” or ”soon,” indicating that the eviction is planned but not yet happend.<br>Few-Shot Examples:<br>- “The landlord has given the tenant a final warning, and eviction proceedings are expected to start next month if the rent is not paid.”<br>- “The tenant has been notified that they must vacate the premises in the coming weeks due to repeated violations of the lease.”<br>- “Eviction is planned for the near future, as the landlord has expressed intent to reclaim the property due to nonpayment.”<br>- “The landlord mentioned that they will file for eviction soon if the tenant does not comply with the notice to remedy the situation.”<br>- “Next week, the landlord plans to serve an eviction notice due to ongoing illegal activities on the property.”
Eviction_mr_current	1. Mutual Rescission (mr) refers to a specific legal agreement in which both the landlord and tenant agree to terminate the lease early. This occurs after eviction proceedings have started, but the eviction process has not yet reached its final stage. As a result of this agreement, the tenant voluntarily vacates the rental property, and the eviction process is stopped before completion. All necessary legal proceedings are concluded, and the tenant no longer has access to the property.<br>- Consideration: When generating the note, it should mention that the Mutual Rescission has already taken place, the eviction process had already stopped, and the tenant is no longer living at or has access to the property.<br>2. Current means that the agreement or action related to mutual rescission is mentioned within a recent period (e.g., ”a few months ago,” ”recently,” ”this year”).
Eviction_mr_history	1. Mutual Rescission (mr) refers to a specific legal agreement in which both the landlord and tenant agree to terminate the lease early. This occurs after eviction proceedings have started, but the eviction process has not yet reached its final stage. As a result of this agreement, the tenant voluntarily vacates the rental property, and the eviction process is stopped before completion. All necessary legal proceedings are concluded, and the tenant no longer has access to the property.<br>- Consideration: When generating the note, it should mention that the Mutual Rescission has already taken place, the eviction process had already stopped, and the tenant is no longer living at or has access to the property.<br>2. History refers to events related to eviction that happened in the past, but with less specificity about the timing. This includes cases where the Mutual Rescission occurred in the distant past (e.g., ”last year,” ”several years ago,” or ”a few years ago”), or where the mutual rescission agreement or eviction action itself isn’t explicitly stated.<br>- Consideration: The note should mention that the event was in the past, providing some general time reference, but without specifying the exact date or events.
Eviction_absent	The text clearly states ”never evicted” or ”no history of eviction”.

Table S6. Eviction-related Class Definitions

SDoH	Definition (/Examples)
Homelessness	An individual or family who lacks a fixed, regular, and adequate nighttime residence, such as those living in emergency shelters, transitional housing, or places not meant for habitation.<br>Here are some few-shot examples: "...is homeless and lives in a shelter…", "...contact one of the homeless shelters....", "...found it difficult to secure housing and ended up living in his car…", "...relying on friends and temporary shelters for support…", "...is actively seeking employment and more permanent housing but has faced numerous obstacles…", "living on the streets…", "...living in a homeless encampment...", "...couch surfing..."
InadequateHousing	Inadequate housing is defined as an occupied housing unit that has moderate or severe physical problems (e.g., deficiencies in plumbing, heating, electricity, hallways, and upkeep). Examples of moderate physical problems in a unit include two or more breakdowns of the toilets that lasted more than 6 months, unvented primary heating equipment, or lack of a complete kitchen facility in the unit. Severe physical problems include lack of running hot or cold water, lack of a working toilet, and exposed wiring.<br>Here are some few-shot examples: "...lives in an old apartment building with severe structural issues...", "...live in an apartment that lacks a functioning heating system…", "...a family, consisting of six members spanning three generations, lives in a cramped two-bedroom apartment…", "...unsafe housing situation…", "...unsanitary living conditions…", "...polluted living environment…", "...lead and toxic exposures in home…"
LackOfAdequateFood	Food insecurity is the limited or inadequate access to food because of insufficient money and other resources for food. Food security, at the individual, household, national, regional, and global levels [is achieved] when all people, at all times, have physical and economic access to sufficient, safe, and nutritious food to meet their dietary needs and food preferences for an active and healthy life. This definition suggests that food insecurity is the absence of one or more of these conditions.<br>Here are some few-shot examples: "...lack the variety and nutrients... ", "...frequently goes hungry or eats whatever is available...", "...no supermarkets...", "...difficult to access better food sources outside the neighborhood...", "...uses food pantries/soup kitchens for food…", "...does not have stable food sources…", "...lives in a food desert…"
FinancialInsecurity	Economic insecurity can be defined as "the anxiety produced by the possible exposure to adverse economic events and by the anticipation of the difficulty to recover from them". Examples could include a fear of unemployment, an expectation of a worsening financial situation, money mismanagement, or being financially exploited or a victim of financial scam.<br>Here are some few-shot examples: "...has been having a lot of stress recently due to financial concerns.", "...was very concerned about the financial burden of hospitalization, medications and potential surgery.", "Rising living costs, including healthcare and housing, have made it difficult for them to cover their monthly expenses.", "The irregular income and lack of benefits make it difficult to budget and plan for the future.", "...has experienced several months of financial difficulty due to job loss.", "...not managing money well…", "...lacks financial literacy…", "...mismanaging funds.", "...lack of stable income…"
HousingInstability	Housing instability is variably defined as having difficulty paying rent, spending more than 50% of household income on housing, having frequent moves, living in overcrowded conditions, or doubling up with friends and relatives. Unstably housed, housing insecure, or in a temporary housing situation. At risk of being homeless or at imminent risk of being homeless.<br>Here are some few-shot examples: "...has moved three times...", "…fell behind on his rent payments...", "...live together with three families to share...", "...couch surfing…", "...temporarily staying with friends/family…", "...staying in a motel/hotel…", "...in a temporary housing situation…", "...at risk of losing their housing…"
MaterialHardship	Material hardships, defined as difficulty meeting basic needs such as food, housing or medical care, are common among low-income households.<br>Here are some few-shot examples: "Their electricity was cut off because they couldn't make the payments.", "...cannot afford to buy winter coats or shoes that fit correctly.", "...cannot afford the necessary school supplies and textbooks for their three children.", "...cannot afford to participate in health and wellness activities such as exercise classes, which are essential for managing his chronic conditions."
TransportationInsecurity	Transportation insecurity occurs when a person is regularly unable to get from place to place in a safe or timely manner because of a lack of resources. This can limit a person's access to work, school, medical care, social activities, and more.<br>Here are some few-shot examples: "…lives in a rural area where there are no public transportation options. The nearest town with essential services such as grocery stores, schools, and healthcare facilities is 20 miles away.", "Buses and trains are often delayed or overcrowded, making it difficult for her to get to work on time.", "...can't afford the transportation fare, he and his children have to walk long distances, often in unsafe conditions.", "...uses a wheelchair and often finds that public transportation in her city is not fully accessible.", "...does not own a car…", "...does not have bus passes…"

Table S7. Non-Eviction-related Class Definition

Instruction / Prompt	Detail
Augmenting Prompt	You are tasked with rewriting the raw notes to become related to some SDoH label.<br>Here is the raw note: *raw_notes*<br>And the specific label: *label*<br>The definition of the label: *definition*<br>Note:<br>1. Do NOT include the definitions or examples provided in your rewrited content.<br>2. Focus exclusively on the social history, disregarding sentences related to family history or other topics.<br>3. The augmented notes should clearly reflect the label context, be contextually coherent, with varied and diverse expressions.<br>4. The augmented note should be a detailed description of a specific patient case that illustrates the application of the SDoH label. Focus on the unique circumstances, events, and actions related to this individual case. Avoid using general or broad descriptions of processes or procedures; instead, provide concrete details and examples that are directly relevant to the patient's situation.<br>5. Your output should not exceed 100 words.<br>Augmented Notes:<br><br>input_variables=["raw_notes", "label", "definition"]
Annotation Prompt in Step 1: Binary Classification	Go through each sentence of the patient note. If a sentence reflects eviction-related social determinants of health (SDoH), assign the label "Yes", else annotate as label "No"
Annotation Prompt in Step 2: Eviction Multi-Class	Go through each sentence of the patient note. If a sentence reflects eviction-related social determinants of health (SDoH), assign the most appropriate label from the following list: "t3_Eviction_absent", "t3_Eviction_present_history", "t3_Eviction_present_current", "t3_Eviction_pending", "t3_Eviction_mr_history", "t3_Eviction_mr_current", "Other". For status part, if no eviction in the history and in the future: "absent"; if eviction is completed: "present"; if eviction noticed but not completed: "pending"; if eviction might be happend in the future: "hypothetical"; if mutual rescission: "mr". For timeframe part when "present" or "mr" status, if it is happened within this natural year: "current". If not shown specific time or noticed a time before this natural year: "history".
Annotation Prompt in Step 3: Non-Eviction Multi-Class	Choose the most approperate label from "t1_Homelessness", "t1_InadequateHousing", "t1_LackOfAdequateFood", "t2_FinancialInsecurity", "t2_HousingInstability", "t2_MaterialHardship", "t2_TransportationInsecurity", "Other".<br>'t1_Homelessness': An individual or family who lacks a fixed, regular, and adequate nighttime residence, such as those living in emergency shelters, transitional housing, or places not meant for habitation.<br>'t1_InadequateHousing': an occupied housing unit that has moderate or severe physical problems (e.g., deficiencies in plumbing, heating, electricity, hallways, and upkeep)<br>'t1_LackOfAdequateFood': is the limited or inadequate access to food because of insufficient money and other resources for food.<br>'t2_FinancialInsecurity': the anxiety produced by the possible exposure to adverse economic events and by the anticipation of the difficulty to recover from them. Examples could include a fear of unemployment, an expectation of a worsening financial situation, money mismanagement, or being financially exploited or a victim of financial scam.<br>'t2_HousingInstability': having difficulty paying rent, spending more than 50% of household income on housing, having frequent moves, living in overcrowded conditions, or doubling up with friends and relatives. 't2_MaterialHardship': difficulty meeting basic needs such as food, housing or medical care, are common among low-income households.<br>'t2_TransportationInsecurity': occurs when a person is regularly unable to get from place to place in a safe or timely manner because of a lack of resources. This can limit a person's access to work, school, medical care, social activities, and more.
Human Annotation for Augmentation Period	We need to check the quality of the GPT-generated notes to refine our prompts, and this requires manual verification—your role in this project. You'll review the entries, marking them 'True' if correct, and giving feedback if false.<br>We have 14 labels, (including "t1_Homelessness", "t1_InadequateHousing", "t1_LackOfAdequateFood", "t2_FinancialInsecurity", "t2_HousingInstability", "t2_MaterialHardship", "t2_TransportationInsecurity", "t3_Eviction_absent", "t3_Eviction_present_history", "t3_Eviction_present_current", "t3_Eviction_pending", "t3_Eviction_mr_history", "t3_Eviction_mr_current"), which make up our initial data set, with 20 entries for each label. We've engaged three experts to ensure accuracy by cross-verifying the data. Each label is annotated by two different experts, so each expert will annotate over 200 entries.<br>If you are not sure the definitions of some label, you can check the document (Same as Supplementary Table S6 and S7).
Manual Rewrite Notes for Devset	We need your assistance to rewrite clinical notes from MIMIC to reflect specific social determinants of health (SDoH) classes related to eviction. For each note and assigned class/label, please modify the content to clearly demonstrate the corresponding eviction situation while preserving the original MIMIC documentation style. Your rewrites should maintain the clinical tone, formatting, and structure of the original notes (including headers, abbreviations, and documentation patterns typical in MIMIC), but incorporate eviction-related circumstances that align with the specified class (e.g., t3_Eviction_present_history, t3_Eviction_pending, etc.). The goal is to create authentic-looking clinical documentation that can be used to augment our training data for eviction classification models.<br>If you are not sure the definitions of some label, you can check the document (Same as Supplementary Table S6 and S7).
Manual Double-Check Rewritten Notes	We have some data entries related to eviction that were rewritten by medical students. However, since they are not experts, we need your help to double-check their correctness. This is the same as the task you've worked on before—just review and mark each entry as either True or False.

Table S8. Human Instructions / LLM Prompt

4. Results on REC. Adding SynthEHR-Eviction yields consistent Macro-F1 gains across model families on this fully real external dataset. For Bio_ClinicalBERT (the core baseline used in[?]), REC-only achieves 0.60 ± 3.10, and REC + SynthEHR-Eviction achieves 0.64 ± 1.05 ($\Delta = +0.04$). For LLaMA-3.1-8B, Macro-F1 increases from 0.79 ± 2.85 to 0.82 ± 0.52 ($\Delta = +0.03$). For LLaMA-3.2-3B, it increases from 0.77 ± 2.70 to 0.79 ± 0.40 ($\Delta = +0.02$). For Qwen2.5-7B, it increases from 0.80 ± 1.25 to 0.83 ± 0.18 ($\Delta = +0.03$). For Qwen2.5-3B, it increases from 0.78 ± 1.45 to 0.80 ± 0.70 ($\Delta = +0.02$). These gains are modest, but they are systematic, and they show that adding SynthEHR-Eviction improves performance on a real external dataset rather than hurting it.

Training Set	Sample Note
Synth	The patient, a widowed immigrant living with a relative, faces potential housing instability. Having lived in the country for about 50 years, her current housing situation may be threatened, necessitating discussions about mutual rescission to ensure she can voluntarily vacate her rental early if needed, which reflects her vulnerabilities related to social determinants of health.
MIMIC	Social History: + Tob, 1.5 ppy X many years, no EtOH, was forced to remove from the rented house, has a child in their 20s
PMC-Patient	Our patient is a girl aged 10–14 years with an unremarkable medical history, yet her family history is significant for allergies and asthma. She initially presented, to another institute, with multiple pruritic facial skin lesions and a pruritic left intranasal lump. Apart from having two erythematous pruritic plaques in the left suborbital region and a yellowish pruritic lump occupying the left nasal vestibule, her physical examination proved to be insignificant without any lymphadenopathies or salivary gland enlargements. Consequently, a laboratory workup was conducted in addition to an excisional biopsy of one dermatologic lesion and a needle biopsy of the nasal lesion. Both biopsies exhibited nonspecific inflammation with granulation, necrosis, and no signs of malignancy. No specific diagnosis was made. The patient was started on hydrogen peroxide treatment for the skin lesions, which resolved completely with no recurrence. Simultaneously, another lump started growing in the right nasal vestibule. Suspecting an inflammatory etiology, she was started on oral prednisolone 1 mg/kg/day by mouth twice a day. Despite therapy, these nasal lumps continued growing. Hence, prednisolone was discontinued after 10 days of therapy to start an indomethacin trial of 1.5 mg/kg/day by mouth twice a day for 14 days, which was also discontinued due to its inefficacy. Then a decision to perform surgery was taken, and the presurgical computed tomography (CT) scan revealed bilateral soft tissue masses arising from the right and left nasal vestibules. Although she had undergone many surgical attempts to remove the lumps, none of them succeeded and both lumps flared in size. After a couple of months, our patient presented to our institution with bilateral nasovestibular lumps; they were massive in size, occluding nasal entrance and protruding outside the nose (Fig.). We did an extensive laboratory workup to exclude any comorbidities (Table). We did a fine-needle aspiration (FNA) biopsy of the lesion, which was diagnostic of ALHE (Fig.). Our following surgical attempt included complete mass resection. Despite surgery and postsurgical treatment with topical steroid creams, the lesion recurred. Consequently, we started the patient on intralesional prednisolone twice a month and topical 0.1% tacrolimus ointment twice daily. This latter regimen seemed to slightly control the lesion's growth, causing a limited regression in size after 4 months of treatment (Fig.). A timeline of the patient's case can be seen in (Fig.). A family member reported her full adherence to treatment. The patient herself reported decreased quality of life and impaired social interactions due to the disfiguring lesions. She also reported marked fear and distress because of the ineffectiveness of multiple therapeutic regimens and surgeries. A relative reported severe financial burden due to the high costs of the treatments. The patient was evicted from the rented house due to a series of late rent payments recently.

Table S9. Sample Patient Notes Across Different Training Datasets (Age ranges and family relations anonymized)

Algorithm 1: Pipeline for Each Augmenter

Require: Definition of SDoH category D, Raw note N
Ensure: Optimized prompt, Verified augmented notes

Model: LLM
$input\_prompt \leftarrow D$ ▷ Initialize with category definition
while True **do**
    $augmented\_notes \leftarrow \{\}$
    $output \leftarrow$ LLM($input\_prompt$) ▷ Generate notes based on D and N
    for each note n in $output$ **do**
        $verification, feedback \leftarrow$ HumanVerify(n)
        if $verification ==$ True **then**
            $augmented\_notes \leftarrow augmented\_notes \cup \{n\}$
        else
            Save $feedback$
        end if
    end for
    $accuracy \leftarrow \frac{\text{Number of True verifications}}{\text{Total number of notes}}$
    if $accuracy \geq 0.90$ **then**
        break
    else
        $input\_prompt \leftarrow$ OptimizePrompt($input\_prompt$, $feedback$)
    end if
end while
return $input\_prompt$, $augmented\_notes$

Algorithm 2: Pipeline for Annotators

Require: Trainset ($notes, labels$), Devset ($notes, labels$)
Ensure: Trained DSPy models, Classification of SDoH categories

Model: DSPy
Step 1: Binary Classification
$trainset_1 \leftarrow (notes, labels)$ ▷ Eviction-related (Yes/No)
$model_1 \leftarrow$ TrainDSPy($trainset_1$)
$accuracy_1 \leftarrow$ ValidateModel($model_1$, $devset$)
Step 2: Eviction Multi-Class
$trainset_2 \leftarrow (notes, labels)$ ▷ 7 eviction-related classes
$model_2 \leftarrow$ TrainDSPy($trainset_2$)
$accuracy_2 \leftarrow$ ValidateModel($model_2$, $devset$)
Step 3: Non-Eviction Multi-Class
$trainset_3 \leftarrow (notes, labels)$ ▷ 7 non-eviction classes
$model_3 \leftarrow$ TrainDSPy($trainset_3$)
$accuracy_3 \leftarrow$ ValidateModel($model_3$, $devset$)
return $model_1, model_2, model_3$

Table S10. Pipelines for Augmenters and Annotators

SDoH	Keywords
t1_Homelessness	homeless, homelessness, shelter, transitional housing, living in car, living on streets, couch surfing, lacks housing, no fixed residence, no permanent home
t1_InadequateHousing	inadequate housing, structural issues, structural problems, deficiencies in plumbing, heating problems, no heating, electrical problems, lack of running water, broken toilet, no toilet, no kitchen, cramped apartment, overcrowded, unsafe housing, unsanitary living, polluted environment, lead exposure, toxic exposure, mold
t1_LackOfAdequateFood	food insecurity, limited access to food, insufficient food, lacks variety, lacks nutrients, no supermarkets, difficult to access food, unstable food sources, cannot afford food, malnutrition, undernourished, skipping meals, reliance on food assistance
t2_FinancialInsecurity	financial insecurity, economic insecurity, financial concerns, financial stress, financial burden, affordability issues, rising living costs, difficulty covering expenses, budget difficulties, lacks financial literacy, no stable income, debt, financial hardship
t2_HousingInstability	housing instability, difficulty paying rent, frequent moves, multiple moves, families sharing housing
t2_MaterialHardship	material hardship, difficulty meeting basic needs, utilities cut off, cannot afford clothing, winter coats, cannot afford school supplies, cannot afford health activities, limited resources for essentials, unable to afford medications, basic needs not met, lacks essential household items
t2_TransportationInsecurity	transportation insecurity, lack of transportation, no public transportation, transportation issues, cannot afford transportation fare, long walking distances, inaccessible transportation, no car, unreliable transportation, limited mobility, transportation costs prohibitive

Table S11. Keywords

Case	Analysis
Case 1	**Physician Note**: The patient, a retired pathologist, experienced eviction due to ongoing financial strain as a relative battles a chronic illness. Despite his extensive career, their medical expenses have caused significant challenges, so he resides in a group home at [**Hospital1 3494**] now.<br><br>**Ground Truth**: t3_Eviction_present_history<br>**Prediction**(Llama-3.1-8B-FT): t3_Eviction_present_current<br><br>**Analysis**: This misclassification likely stems from temporal ambiguity in the text. While the eviction explicitly occurred with no specific timeframe mentioned (indicating a historical event), the phrase 'resides in a group home at [Hospital1 3494] now' may have caused the model to associate the current housing situation with ongoing eviction issues. This highlights the challenge in distinguishing between historical events and currently active situations, particularly when the specific timing of the eviction is not mentioned and 'now' appears in the context.
Case 2	**Physician Note**: The patient reports a history of housing instability, having faced an eviction process stemming from unpaid rent two months ago. This event has exacerbated her ongoing health issues, such as bilateral lower extremity edema and nocturnal dyspnea, highlighting the interplay between her living situation and overall well-being.<br><br>**Ground Truth**: t3_Eviction_present_current<br>**Prediction**(Llama-3.1-8B-FT): t3_Eviction_present_history<br><br>**Analysis**: In this case, the model misclassified the eviction situation as `present_history` rather than `present_current`. This error likely stems from multiple factors in the text. First, the explicit mention of "reports a history of housing instability" may have primed the model to categorize the entire situation as historical, despite the more recent "two months ago" timeframe which falls within our one-year threshold for `present_current` classification. The lexical cue "history" appears early in the sentence, potentially biasing the model's interpretation of subsequent temporal information. Additionally, phrases like "having faced an eviction process" use past-tense constructions that might further reinforce a historical framing, even though the event occurred recently. This demonstrates how models can be overly influenced by specific terminology like "history" rather than properly processing the actual temporal indicators ("two months ago") in relation to the annotation schema. This challenge highlights the need for models to prioritize explicit timeframes over potentially misleading lexical cues when making temporal classifications according to specific guidelines.
Case 3	**Physician Note**: The patient lives with a relative, who has been recently ill, leading to challenges in meal preparation and unintended weight loss. This situation contributes to financial strain, leading to a current eviction process initiated by their landlord.<br><br>**Ground Truth**: t3_Eviction_pending (rationale='produce the answer. We need to identify any mention of eviction-related social determinants of health (SDoH) in the patient's note. The note describes a situation where the patient and a relative are currently undergoing an eviction process initiated by their landlord due to financial strain. This indicates that the eviction process is active and ongoing. Therefore, the most appropriate label for this situation is "t3_Eviction_pending".')<br><br>**Prediction**(Llama-3.2-3B-FT):<br>**Training set with Reasoning**: t3_Eviction_pending. Output_Reasoning: determine the appropriate label for the patient's eviction status. The fact states that the patient is currently facing an eviction process initiated by their landlord, indicating that eviction proceedings are ongoing. Since the eviction is not yet completed and the patient is still residing in the property while dealing with financial strain, we categorize this situation as "pending."<br>**Training set without Reasoning**: t3_Eviction_present_current<br><br>**Analysis**: This case offers valuable insights into how the presence of reasoning in training data affects both prediction accuracy and reasoning quality. When trained with reasoning annotations, the Llama-3.2-3B-FT model correctly identifies the eviction status as "t3_Eviction_pending." However, when the same model is trained without reasoning, it incorrectly classifies the situation as "t3_Eviction_present_current," failing to distinguish between an active ongoing eviction process (pending) and a completed recent eviction (present_current). Comparing the reasoning produced by the model trained with reasoning to the ground truth reasoning, we observe important similarities and differences. Both correctly identify the key evidence: "eviction process initiated by their landlord." Both reasoning paths also correctly emphasize that this indicates an ongoing, active eviction situation rather than a completed one. However, the model's reasoning is more concise and adds the specific detail that "the patient is still residing in the property," which is implied but not explicitly stated in the ground truth reasoning.

Table S12. Error Analysis - Case 1-3

Case	Analysis
Case 4	**Physician Note**: The individual does not currently use tobacco and has not faced eviction. He drinks up to 10 beers every weekend. He has used cocaine several times a week for the past several years, but has not used any for a month. He faces potential housing instability after mutually agreeing to terminate his lease. Previously retired and supporting troubled youth, the individual now navigates social pressures and community resources while managing his lifestyle choices, including alcohol consumption and past smoking history, which may impact his wellbeing.<br><br>**Ground Truth**: t3_Eviction_mr_current (rationale='produce the answer. We need to identify any mention of eviction-related social determinants of health (SDoH) in the individual's note. The note describes a situation where the individual has mutually agreed to terminate her lease, which indicates a mutual rescission of the lease. This suggests that the eviction process is not adversarial but rather a mutual agreement to end the lease. Therefore, the most appropriate label for this situation is "t3_Eviction_mr_current" as the mutual rescission is happening currently.')<br><br>**Prediction**(Llama-3.1-8B-FT): t3_Eviction_mr_current (Reasoning: determine the appropriate label for the patient's eviction status. The individual has mutually agreed to terminate her lease, indicating that she is currently in a situation where she is not facing eviction in the traditional sense, but rather has made a decision to vacate the property. This mutual rescission of the lease suggests that she is actively navigating her housing situation, but it does not indicate an eviction that has already occurred or is currently in progress. Therefore, we categorize this situation as a mutual rescission of the lease.)<br><br>**GPT-as-judge**: False. The reasoning provided does not fully align with the ground truth. The ground truth clearly states that the situation should be labeled as 't3_Eviction_mr_current' because the mutual rescission of the lease is happening currently. However, the reasoning suggests that the individual is not facing eviction in the traditional sense and does not indicate an eviction that has already occurred or is currently in progress. This implies a misunderstanding of the label 't3_Eviction_mr_current,' which is meant to capture the current mutual rescission of the lease. The reasoning fails to recognize that a mutual rescission is a form of eviction-related SDoH and should be labeled as such. Therefore, there is a gap in understanding the categorization of the situation as per the ground truth.
Case 5	**Physician Note**: The patient lives at home with family members, one of whom is ESRD on HD and another provides care for the patient. They are in process of eviction because the landlord issues a notice due to missed rent payments stemming from financial strain.<br><br>**Ground Truth**: t3_Eviction_pending (rationale='produce the answer. We need to identify any mention of eviction-related social determinants of health (SDoH) in the patient's note. The note describes a situation where the patient and their family are in the process of eviction due to missed rent payments caused by financial strain. This indicates that eviction proceedings have started but are not yet completed. Therefore, the most appropriate label for this situation is "t3_Eviction_pending".')<br><br>**Prediction**(Llama-3.1-8B-FT): t3_Eviction_pending (Reasoning: produce the answer. The facts indicate that the patient is currently facing eviction due to missed rent payments, which have resulted in a notice from the landlord. Since the eviction process has not yet been completed, but is in progress, the status is "pending." The timeframe is not specified as occurring within the current year, so we will label it as "history." Therefore, the appropriate label will reflect that the eviction is not yet finalized, but is actively being pursued.)<br><br>**GPT-as-judge**: False. The reasoning provided does not logically support the ground truth. The ground truth clearly identifies the situation as 't3_Eviction_pending' based on the fact that eviction proceedings have started but are not yet completed. However, the reasoning introduces an additional and incorrect element by labeling the situation as 'history' due to unspecified timing. This contradicts the ground truth, which does not consider the timeframe as a factor for labeling. The correct label should solely reflect the current status of the eviction process, which is 'pending,' without introducing the notion of 'history.' Therefore, the reasoning is incorrect as it deviates from the ground truth by adding an unnecessary and unsupported condition.
Case 6	**Physician Note**: Social History: Lives alone with PCA 20 hours per day. The individual recently faced financial strain, which led to eviction from her rental home due to unpaid rent. Fortunately, a relative lives nearby, so she moved in with the relative.<br><br>**Ground Truth**: t3_Eviction_present_current (rationale='produce the answer. We need to identify any mention of eviction-related social determinants of health (SDoH) in the patient's social history. The note indicates that the individual recently faced financial strain, leading to an eviction from her rental home due to unpaid rent. She moved to her relative's home, which suggests that the eviction process was completed, and she found an alternative living arrangement. Therefore, the most appropriate label for this situation is "t3_Eviction_present_current")<br><br>**Prediction**(Llama-3.1-8B-FT): t3_Eviction_present_current (Reasoning: determine the appropriate label for the patient's eviction status. The fact states that the individual is currently facing financial strain and has not paid rent, which has led to an eviction notice. Since the eviction process has not yet been completed but is in progress, we categorize this situation as "present." Additionally, the individual is currently living with a relative, indicating a temporary housing solution, but the ongoing eviction status suggests that the situation is still precarious.)<br><br>**GPT-as-judge**: False. The reasoning provided does not logically support the ground truth. The ground truth indicates that the eviction process was completed, as the individual moved to a relative's home after being evicted due to unpaid rent. This situation is labeled as 't3_Eviction_present_current,' which implies that the eviction has already occurred and the individual is currently dealing with its consequences. However, the reasoning suggests that the eviction process is still in progress and categorizes the situation as 'present,' which contradicts the ground truth. The reasoning fails to acknowledge that the eviction has been completed and misinterprets the current living situation as temporary rather than a result of completed eviction.

Table S13. Error Analysis - Case 4-6

**Models**	**Synth**	**Mimic**	**PMC**	**Avg**
**Best Performance Results (Micro-F1) of Various Models for Three-Step Sequential Task Evaluation**				
GPT-4o-mini	0.518	0.338	0.521	0.459
GPT-4o-mini-DSPy	0.743	0.600	0.736	0.693
GPT-4o	0.721	0.650	0.579	0.650
GPT-4o-DSPy (baseline)	0.904	0.862	0.857	0.874
bert_base_cased-FT	0.893	0.754	0.600	0.749
biobert-v1.1-FT	0.896	0.735	0.621	0.751
Bio_ClinicalBERT-FT	0.886	0.592	0.421	0.633
Llama-3.1-8B-FT	0.896	0.873	0.829	0.866
Llama-3.2-3B-FT	0.882	0.827	0.736	0.815
Qwen2.5-7B-FT	0.907	0.877	0.814	0.866
Qwen2.5-3B-FT	0.893	0.815	0.750	0.819
**Overall Performance**	**0.911**	**0.885**	**0.866**	**0.887**

Table S14. The Best Performance Results (accuracy) for each model is calculated by considering a prediction correct only when step 1 is correct, and both step 2 and step 3 are correct. Finally, the Overall Performance is calculated by considering a prediction correct only when step 1 (performed by GPT-4o-DSPy) is correct, and both step 2 and step 3 (performed by LLaMA-3.1-8B-FT) are correct.

**Model**	**Step2 Eviction Micro-F1**	**Step2 Eviction Macro-F1**	**Step3 Non-eviction Micro-F1**	**Step3 Non-eviction Macro-F1**
Llama-3.2-3B	0.042	0.037	0.143	0.231
Llama-3.1-8B	0.277	0.197	0.543	0.454
Qwen2.5-3B	0.201	0.142	0.267	0.242
Qwen2.5-7B	0.317	0.297	0.575	0.543

Table S15. Zero-shot performance (training size = 0) for open-source LLMs.
